# *De novo* assembly of *Aureococcus anophagefferens* transcriptomes reveals diverse responses to the low nutrient and low light conditions present during blooms

**DOI:** 10.3389/fmicb.2014.00375

**Published:** 2014-07-24

**Authors:** Kyle R. Frischkorn, Matthew J. Harke, Christopher J. Gobler, Sonya T. Dyhrman

**Affiliations:** ^1^Department of Earth and Environmental Sciences and the Lamont-Doherty Earth Observatory, Columbia UniversityPalisades, NY, USA; ^2^School of Marine and Atmospheric Sciences, Stony Brook UniversitySouthampton, NY, USA

**Keywords:** *Aureococcus anophagefferens*, phytoplankton, harmful algal bloom, eco-physiology, transcriptome, *de novo* assembly, gene expression

## Abstract

Transcriptome profiling was performed on the harmful algal bloom-forming pelagophyte *Aureococcus anophagefferens* strain CCMP 1850 to assess responses to common stressors for dense phytoplankton blooms: low inorganic nitrogen concentrations, low inorganic phosphorus concentrations, low light levels, and a replete control. The *de novo* assemblies of pooled reads from all treatments reconstructed ~54,000 transcripts using Trinity, and ~31,000 transcripts using ABySS. Comparison to the strain CCMP 1984 genome showed that the majority of the gene models were present in both *de novo* assemblies and that roughly 95% of contigs from both assemblies mapped to the genome, with Trinity capturing slightly more genome content. Sequence reads were mapped back to the *de novo* assemblies as well as the gene models and differential expression was analyzed using a Bayesian approach called Analysis of Sequence Counts (ASC). On average, 93% of significantly upregulated transcripts recovered by genome mapping were present in the significantly upregulated pool from both *de novo* assembly methods. Transcripts related to the transport and metabolism of nitrogen were upregulated in the low nitrogen treatment, transcripts encoding enzymes that hydrolyze organic phosphorus or relieve arsenic toxicity were upregulated in the low phosphorus treatment, and transcripts for enzymes that catabolize organic compounds, restructure lipid membranes, or are involved in sulfolipid biosynthesis were upregulated in the low light treatment. A comparison of this transcriptome to the nutrient regulated transcriptional response of CCMP 1984 identified conserved responses between these two strains. These analyses reveal the transcriptional underpinnings of physiological shifts that could contribute to the ecological success of this species *in situ*: organic matter processing, metal detoxification, lipid restructuring, and photosynthetic apparatus turnover.

## Introduction

In 1985 coastal embayments around Long Island, New York were disrupted by dense phytoplankton blooms that colored the water a murky brown (Gobler et al., 2005; Gobler and Sunda, 2012). Researchers identified the causative organism of these “brown tide” blooms as the pelagophyte phytoplankton *Aureococcus anophagefferens.* In the years since the first *A. anophagefferens* bloom, these brown tides have recurred annually and now extend down the eastern seaboard as far as Virginia (Kana et al., 2004; Gobler et al., 2005; Boneillo and Mulholland, 2013). In recent years, blooms of *A. anophagefferens* have also been detected off the coast of South Africa (Gobler et al., 2005) and in China (Zhang et al., 2012). Brown tides do not pose a direct threat to human health, however they do have damaging ecosystem effects. Brown tides have also been linked to the collapse of fisheries due to *A. anophagefferens'* toxicity to shellfish and the elevated cell densities during blooms can attenuate light and destroy seagrass beds (Gobler et al., 2005).

Brown tides occur in waters in which the concentration of organic carbon and organic nitrogen are high relative to inorganic nutrients and heightened cell densities attenuate light in the water column (Anderson et al., 2002; Sunda et al., 2006; Heisler et al., 2008; Gobler et al., 2011). These observations have been corroborated with laboratory studies that show *A. anophagefferens* can grow using organic compounds as a sole nutrient source (Berg et al., 2008; Wurch et al., 2011b) and can survive prolonged periods in the dark (Popels and Hutchins, 2002). Sequencing and analysis of the CCMP 1984 genome revealed the presence of a number of genes that were hypothesized to contribute to *A. anophagefferens* success in bloom conditions. For example, relative to other co-occurring phytoplankton, the *A. anophagefferens* genome contains more light harvesting complex proteins as well as numerous genes implicated in the import and breakdown of diverse organic compounds including dissolved organic nitrogen (DON) and dissolved organic phosphorus (DOP) (Gobler et al., 2011). Low resolution (Serial Analysis of Gene Expression) transcriptional studies and targeted approaches focused on nitrogen suggest that *A. anophagefferens* strain CCMP 1984 upregulates genes related to the utilization of a diverse array of DON and DOP sources in low inorganic nutrient concentrations (Berg et al., 2008; Wurch et al., 2011b). Despite these advances, the physiological mechanisms that facilitate growth in the low nitrogen (N), low phosphorus (P), and low light conditions found during blooms are still poorly understood. Further, it remains unclear if these capabilities are shared across strains or if they contribute to strain-level niche differentiation in the environment. Strain differentiation is of particular interest in *A. anophagefferens* as some strains are acutely toxic to bivalves and copepods, while others are not (Harke et al., 2011).

High throughput transcriptome sequencing can be used to reveal the system-wide response of an organism to experimentally controlled perturbations, with or without a genome (Grabherr et al., 2011). Here, the transcripts from cultures of the toxic *A. anophagefferens* strain, CCMP 1850, grown under low N, low P, low light, and replete nutrient and light levels were sequenced as part of the Marine Microbial Eukaryote Transcriptome Sequencing Project (MMETSP) (Keeling et al., 2014). Although it is common to map reads to a genome for expression analysis, this can arguably obscure strain heterogeneity. Additionally, the majority of eukaryotic phytoplankton, including the hundreds of genera analyzed through the MMETSP, do not have sequenced genomes. Herein sequenced reads were examined with three different methods: two *de novo* sequence assembly pipelines using the Trinity program and the ABySS program, and one pipeline in which reads were examined by mapping directly to the *A. anophagefferens* CCMP 1984 gene models. In each of the three methods, transcriptomes were also analyzed for significant differential expression between treatments and control using a stringent empirical Bayes method called Analysis of Sequence Counts (ASC) (Wu et al., 2010). ASC is optimized for use without sequenced replicates and has been shown to perform similarly, but conservatively, when compared to other differential expression analyses using a sample dataset with and without sequenced replicates (Wu et al., 2010).

## Materials and methods

### Experimental design

Experiments were performed with *A. anophagefferens* strain CCMP 1850 (isolated from Great South Bay, Long Island, New York, USA, May, 1998) maintained on modified GSe medium (60 μM NH_4_, 5 μM PO_4_) (Doblin et al., 1999) made from 0.2 μm filtered seawater collected from the coastal Atlantic Ocean (final salinity 32). The culture used was not axenic, but was uni-algal and uni-eukaryotic. As a precautionary measure, an antibiotic and antimycotic solution (final concentrations = 100 IU mL^−1^ penicillin, 100 μg mL^−1^ streptomycin, and 0.25 μg mL^−1^ amphotericin B; Mediatech, Inc.) was added to the culture medium immediately before inoculation of cells at a 0.5% concentration (final volume). Experiments were conducted at 21°C on a 14:10 light:dark cycle illuminated by a bank of fluorescent lights at an intensity of 100 μE m^−2^ s^−1^, unless otherwise noted.

Culture conditions included low P (1 μM PO_4_), low N (30 μM NH_4_), low light (20 μE m^−2^ s^−1^), and a culture with replete amounts of N, P, (60 μM NH_4_, 5 μM PO_4_) and light (100 μE m^−2^ s^−1^) to serve as a control. Triplicate cultures were inoculated with 3.5 × 10^5^ cells mL^−1^ and monitored for cell densities, *in vivo* chlorophyll *a* fluorescence, photosynthetic efficiency (Fv/Fm), alkaline phosphatase activity (APA), and dissolved nutrient concentrations. Measurements were made at the same time each day to avoid diel changes in gene expression and cell physiology. The control was harvested during exponential phase growth and the treatments were harvested at the onset of stationary phase to assure limitation by either light, N or P. This approach allows for the identification of N, P, and light effects independently, but common stress responses across all treatments cannot be distinguished from growth rate effects. A similar sampling scheme has been used for previous gene expression studies (e.g., Dyhrman et al., 2006; Wurch et al., 2011b; Dyhrman et al., 2012; Bender et al., 2014).

### Culture analysis

Lugol's iodine preserved cells were enumerated using a Beckman Coulter Multisizer™ 3 Coulter Counter® with a 50 μm aperture (Harke et al., 2011). Nutrient samples were filtered through 0.2 μm polycarbonate filters, and stored frozen. Nitrate was analyzed by reducing the nitrate to nitrite using spongy cadmium as per Jones (1984). Ammonium and phosphate were analyzed using techniques modified from Parsons and colleagues (1984). These nutrient analyses provided 100 ± 10% recovery of standard reference material (SPEX CertiPrep™) for nitrate, ammonium, and phosphate. Bulk APA was measured for each replicate experimental sample on a Turner Designs TD-700 fluorometer (EM filter of 410–600 nm and EX filter of 300–400 nm) using 4-Methylumbelliferone phosphate (250 μ M concentration) as the substrate (Hoppe, 1983). Maximum quantum efficiency of photosystem II (PSII) was estimated from *in vivo* (F_v_) and DCMU (3,4-dichlorophenyl-1,1-dimethylurea)-enhanced *in vivo* fluorescence (F_m_) of each replicate experimental sample on a Turner Designs TD-700 fluorometer (EM filter of >665 nm and EX filter of 340–500 nm). All readings were blank corrected using GSe media. DCMU blocks electron transfer between PSII and PSI and yields maximal fluorescence.

### RNA isolation

At the time of harvest, aliquots of each replicate in each treatment were centrifuged for 10 minutes at 1300 × g at 21°C. The supernatant was decanted and resulting cell pellet was resuspended with 1 mL of remaining media and placed into a 2 mL microcentrifuge tube. The concentrated sample was centrifuged again for 10 min at 1300 × g at 21°C and immediately flash frozen in liquid nitrogen and stored at −80°C. The entire harvest process took <30 min per experimental flask, and was similar to other studies with *Aureococcus* (Wurch et al., 2011a,b, 2014). Total RNA was extracted with a MO BIO UltraClean™ Plant RNA Isolation Kit according to the manufacturer's instructions. A second DNase step was conducted to remove any residual genomic DNA remaining on the spin filters using an Ambion Turbo DNA-*free*™ kit according to the manufacturer's instructions. Cell pellets for each replicate were extracted individually through separate columns and pooled. Sequencing of biological replicates was not available through the MMETSP; in order to account for biological variability in transcriptional response, extracts from triplicate cultures of each treatment and the control were pooled prior to sequencing at the National Center for Genome Resources (NCGR, Santa Fe, NM).

### RNA library preparation and sequencing

Samples were quantified using Invitrogen Qubit Q32855 and RNA quality was assessed using the Agilent 2100 Bioanalyzer. The Illumina TruSeq RNA Sample Preparation Kit was used to generate libraries using ~2 μg of RNA. Sequencing of 50 base pair paired-end reads from each library was performed on an Illumina HiSeq 2000 at the NCGR. Over 2 Gbp of sequence was generated per library. Sequence data is available on the Community Cyberinfrastructure for Advanced Microbial Ecology Research and Analysis (CAMERA) server (http://camera.calit2.net/) with the identification numbers MMETSP0914, MMETSP0915, MMETSP0916, and MMETSP0917.

### Assembly and annotation

Reads were analyzed with three different approaches: mapping reads to the *A. anophagefferens* CCMP 1984 gene models, and two *de novo* assembly pipelines, one utilizing the Trinity *de novo* assembly suite (Grabherr et al., 2011) and the other performed by the NCGR as part of the MMETSP (Keeling et al., 2014) utilizing the ABySS assembler (Birol et al., 2009).

For the ABySS method, the assembly and quantifications provided by NCGR were carried out as follows. Sequences were trimmed using SGA Preprocess at Q15 with the swinging average setting (Simpson and Durbin, 2012). Following trimming, reads shorter than 25 nucleotides were discarded, all treatments were merged and then assembled using ABySS and 20 runs with k-mer sizes ranging from *k* = 26 to *k* = 50 (Simpson et al., 2009). Contigs from all runs were merged and then clustered at 98% sequence identity using CD-Hit (Li and Godzik, 2006). Clustered contigs were then assembled into longer sequences using CAP3 (Huang, 1999) and the paired-end scaffolding feature of ABySS (Simpson et al., 2009). GapCloser from the SOAP *de novo* assembly software was used to close gaps created during scaffolding (Li et al., 2008). Finally, scaffolds were clustered with CD-Hit a second time at 98% identity and sequences less than 150 base pairs were removed (Li and Godzik, 2006).

Prior to commencing the Trinity method, raw reads were trimmed using Trimmomatic with paired-end parameters, sliding window of 6 through 20, and a minimum accepted length of 25 base pairs (Lohse et al., 2012), then reads from all 4 treatments were merged and assembled using the paired end settings of the Trinity software package (Grabherr et al., 2011). Geneious was used to predict open reading frames (ORFs) greater than 100 base pairs with the options to exclude interior sequences and potential outside start/stop codons (www.geneious.com). Clustering of the Trinity assembly was performed using the CD-Hit program (Fu et al., 2012). The resulting sequences were filtered to remove contigs less than 200 base pairs in length.

Transcripts identified by mapping (see below) to the genome were annotated by retrieving annotations from the CCMP 1984 genome (Gobler et al., 2011). Coding sequences in the ABySS assembly were identified using ESTScan (Iseli et al., 1999; Lottaz et al., 2003). Hits against the UniProtKB and Swiss-Prot databases were determined using BLASTp (Altschul et al., 1990). Identified coding sequences were further characterized with Pfam-A, TIGRFAM, and SUPERFAMILY databases using HMMER3 (Haft et al., 2001; Gough et al., 2001; Zhang and Wood, 2003; Bateman, 2004). Trinity assembly contigs were then annotated by aligning against the ABySS assembly contigs; all contigs generated from the Trinity pipeline were additionally compared against the non-redundant protein database on NCBI using BLASTx from the BLAST+ software with an e-value cutoff of 1 × 10^−5^ (Camacho et al., 2009). Trinity method contigs were further annotated by identifying biochemical pathways using the Kyoto Encyclopedia of Genes and Genomes database using the partial genome single-directional best hit method (Kanehisa, 2006). The annotations of the transcripts presented herein are putative, as the functions of these transcripts have not been experimentally verified in *A. anophagefferens* CCMP 1850.

In the *de novo* pipelines, counts for each contig quantified by first using Bowtie 2 run with the “–sensitive” parameters in “–end-to-end” mode in order to align trimmed reads to the final contigs produced by the ABySS or Trinity assemblies. These Bowtie 2 settings were chosen because previous studies have shown that increasing the sensitivity does not yield a dramatic increase in the percentage of reads aligned but does greatly increase computation time (Langmead and Salzberg, 2012) and because Bowtie 2 alignments have been shown to be less sensitive to altered parameters than similar alignment programs (Lindner and Friedel, 2012). The HTSeq Count program was then used to obtain counts (www.huber.embl.de/users/anders/HTSeq/).

For the genome mapping method, TopHat with parameters of 15 threads and an expected inner distance between mate pairs of 100 bp (Roberts et al., 2012) was used to map raw reads from each treatment and the control to the CCMP 1984 genome (genome.jgi.doe.gov/Auran1/Auran1.home.html, Gobler et al., 2011) with 2 mismatches allowed. Following genome mapping, transcript abundance was quantified using HTSeq Count. The *de novo* assemblies from Trinity and ABySS were compared against one another and the CCMP 1984 gene models and the full genome scaffolds by performing reciprocal BLASTn searches with e-value cutoffs of 1 × 10^−5^ (Camacho et al., 2009).

### Expression analysis

For all three pipelines, significant differential expression patterns in the low N, low P, and low light treatments vs. the replete condition control were assigned using a method called ASC (Wu et al., 2010). ASC is an empirical Bayes method that estimates the prior distribution by modeling biological variability using the data itself, rather than imposing a negative binomial distribution. ASC has been shown to perform similarly, but conservatively, relative to other differential expression analyses implemented on data sets with and without replicates (Wu et al., 2010). ASC has been successfully utilized in a number of studies for which sequenced replicates were not available (Alexander et al., 2012; Dyhrman et al., 2012; Thomas et al., 2012; Konotchick et al., 2013). In each condition relative to the control, genes upregulated or downregulated with a fold change greater than or equal to 2 and a posterior probability greater than 0.95 were deemed significantly differentially regulated, which are parameters used in previous studies of this type (Dyhrman et al., 2012).

In the low N treatment, several of the expected *A. anophagefferens* transcriptional responses identified in previous studies (Berg et al., 2008; Wurch et al., 2011b) were not found among the significant transcripts. We compiled a list of significantly upregulated, biologically important low N response genes previously identified in *A. anophagefferens* CCMP 1984 (Berg et al., 2008; Wurch et al., 2011b), as well as other phytoplankton including diatoms (Allen et al., 2011; Bender et al., 2014) and the coccolithophore *Emiliania huxleyi* (Dyhrman et al., 2006). This list included ammonium, amino acids, nitrite, and urea transporters, a xanthine/uracil/vitamin C permease, and the enzymes formamidase, urease, nitrate reductase, and arginase. All transcripts with these functional annotations were selected from within the assembled CCMP 1850 transcriptome and expression patterns across all treatments were examined by plotting the average variance from the normalized count (reads or tags per million; TPM). This expression pattern was compared against the mean variance in TPM from all ASC-identified significantly upregulated low N transcripts in each treatment.

## Results

### Differential growth among treatments

**Figure 1:**
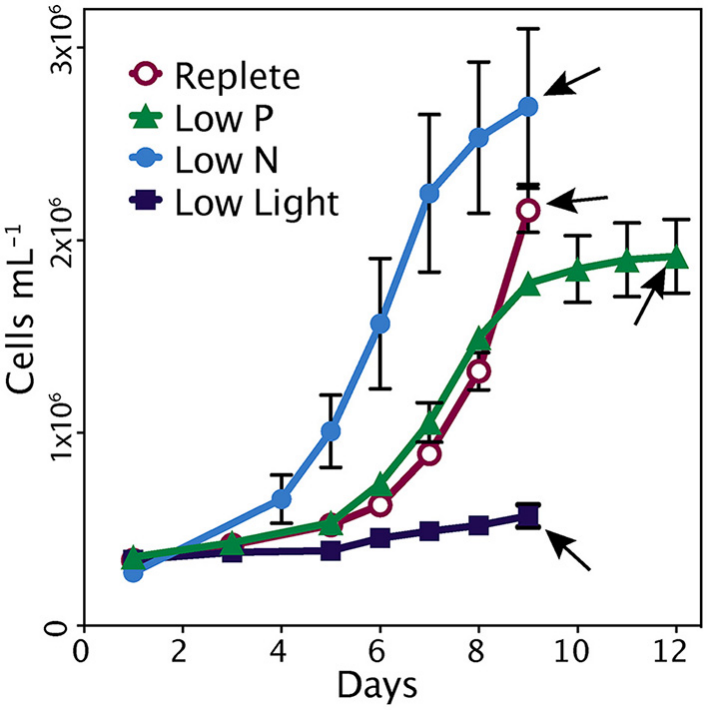
**Cell growth in each treatment**. Error bars represent standard deviation of triplicate cultures. Arrows indicate time of harvest.

The replete cultures were harvested during the exponential phase of growth on day 9 (Figure 1). The low nutrient cultures were harvested in early stationary phase; this corresponded to day 9 in low N and day 12 in low P. The cultures grown in low light maintained a steady low growth rate and were harvested on day 9 (Figure 1). At the time of harvest, control cultures were growing at significantly higher rates (0.49 d^−1^) than those of the treatments, which ranged from 0.01 to 0.09 d^−1^ (*p* < 0.001). The longer lag time in replete and low P cultures relative to low N is likely due to the initial excess of ammonium hindering growth, which has been seen in similar studies (Wurch et al., 2014). In the low N and low P cultures, the reduced growth rates were consistent with reduced concentrations of N or P, respectively (Table 1). Inorganic phosphate concentrations in the low P treatment at the time of harvest were 1.34 ± 0.23 μM and were significantly lower than the other treatments (>3 μM; *p* < 0.001). Nitrate levels were below the detection limit in the low N treatment but were >9 μM in the replete, low P and low light treatments. Ammonium levels in the low N treatment were also significantly lower (1.76 ± 0.28 μM) than the other three treatments (13–38 μM; *p* < 0.001). Photosynthetic efficiency of photosystem II (Fv/Fm) at the time of harvest was significantly higher in the control (0.58 ± 0.02; *p* < 0.05) than all other treatments (Table 1). The APA was measured during stationary phase and just prior to harvesting (days 10 and 11) in the low P cultures, and on days 6 and 7 during exponential phase in the replete condition cultures (Figure 1). The average activity in the triplicate cultures over the 2 days prior to harvesting was 4.0 nmol P L^−1^ h^−1^ in the low P treatment and 2.1 nmol P L^−1^ h^−1^ in the replete cultures.

**Table 1:** **Nutrient concentrations in the culture media and cellular photosynthetic efficiency (Fv/Fm) at the time of harvest**.

	**Replete**	**Low P**	**Low N**	**Low light**
NH_4_ [μM]	37.54 (1.55)	38.03 (0.53)	1.76 (0.28)	13.66 (0.83)
NO_x_ [μM]	10.97 (0.36)	11.01 (0.32)	BD	9.03 (1.50)
PO_4_ [μM]	6.99 (1.55)	1.34 (0.23)	3.64 (0.62)	3.03 (0.10)
Fv/Fm	0.58 (0.02)	0.51 (0.01)	0.47 (0.00)	0.51 (0.01)

The values represent the mean value across 3 replicates with standard deviations in parentheses. BD, below the limit of detection.

### RNA sequencing and *de novo* assembly

Illumina sequencing of the replete sample library yielded 22,291,148 paired reads, low N yielded 23,413,795 paired reads, low P yielded 27,635,028 paired reads, and low light yielded 22,259,313 paired reads. After trimming, the replete library had 21,013,910 paired reads, low N had 20,712,739 paired reads, low P had 25,555,090 paired reads, and low light had 20,840,187 paired reads. Greater than 90% of trimmed reads mapped to the CCMP 1984 genome or *de novo* assemblies in all cases (Table 2). The Trinity assembly yielded 53,886 contigs (N50 = 2148) while the ABySS assembly yielded 31,473 (N50 = 1393) (Table 3). In the Trinity and ABySS assemblies, 85 and 74% of contigs respectively contained ORFs greater than 100 base pairs. Each *de novo* assembly was assessed by comparing the assemblies against one another and against the CCMP 1984 gene models and genome (Gobler et al., 2011). Roughly 98.2% (30,933 out of 31,473) of the ABySS contigs were present in the Trinity assembly, while 85.5% of the Trinity contigs (46,055 out of 53,866) were present in the ABySS assembly (Table 3). BLAST alignments of the *de novo* assemblies against the CCMP 1984 gene models resulted in 8221 out of 11,501 gene models recovered by the Trinity assembly and 7543 out of 11,501 gene models recovered by the ABySS assembly (Table 3). There were 7275 shared gene models between the *de novo* assemblies, with 946 gene models represented solely in the Trinity assembly, and 267 gene models represented solely in the ABySS assembly. However, over 95% of both Trinity and ABySS contigs aligned successfully to the full *A. anophagefferens* CCMP 1984 genome scaffolds. This is a metric that has previously been used to assess the efficacy and relative performance of *de novo* assembly pipelines (Grabherr et al., 2011). The high percentage of contigs aligned to the genome scaffolds relative to gene models recovered is likely due to the presence of multiple isoforms, as multiple isoforms were observed aligning to each gene model. Additionally, the difference could in part be affected by genes missed during modeling as was case with the VTC4 polyphosphate polymerase, discussed below.

**Table 2:** **The percentage of trimmed reads that mapped to the CCMP 1984 gene models and the two CCMP 1850 *de novo* assemblies**.

	**Replete**	**Low N%**	**Low P%**	**Low light%**
1984 gene models	97.8	90.1	93.3	94.1
1850 Trinity assembly	91.8	93.1	93.0	93.3
1850 ABySS assembly	93.9	94.6	95.7	95.0

Values were determined using Bowtie 2 with the “–sensitive” parameter (Langmead and Salzberg, 2012).

**Table 3:** **Trinity and ABySS *de novo* assembly statistics and comparisons between the CCMP 1984 gene model sequences and the assembled contigs from CCMP 1850**.

	**Sequences/contigs**	**Min length**	**Max length**	**Mean length**	**N50**
1984 gene models	11501	150	45147	1601	2340
1850 Trinity assembly	53886	201	20608	1330.5	2148
1850 ABySS assembly	31473	150	16590	891.5	1393
	vs. Trinity^*^	**vs. ABySS**	**1984 full genome coverage**	Gene models recovered^**^	
1984 gene models	82%	76.8%	–	10817	
1850 Trinity assembly	–	85.5%	95.5%	8221	
1850 ABySS assembly	98.2%	–	95.0%	7543	

* Comparisons between the de novo assemblies were made by performing reciprocal BLAST alignments with an e-value cutoff of 1 × 10^−5^ (Camacho et al., 2009).

** Coverage of the CCMP 1984 genome and gene models (Gobler et al., 2011) was tabulated with BLAST using the same parameters described above. The Gene Models Recovered column refers to the number of unique CCMP 1984 gene models found in each assembly.

Clustering the Trinity assembly at 98, 95, and 90% sequence identity reduced the number of contigs to 49,047, 42,508, and 37,314, respectively. Clustering the Trinity assembly at 90% sequence identity did not appreciably decrease the percentage of successfully mapped sequences when compared with the ABySS-generated transcripts: 98% or 30,858 out 31,473 ABySS transcripts mapped to the 37,314 Trinity 90% clusters. The number of gene models represented in the Trinity 90% clusters was also similar to the unclustered Trinity assembly: 8149 out of 11,501 models. Roughly 52% of the total contigs in the clustered Trinity assembly remained unannotated after the annotation steps. Clustering at 90% created a more manageable dataset; this technique has been implemented in past studies as a way to remove redundant sequences (Suzek et al., 2007) and to remove biases caused by abundant sequences (Holm and Sander, 1998).

### Identifying differentially expressed transcripts

**Figure 2:**
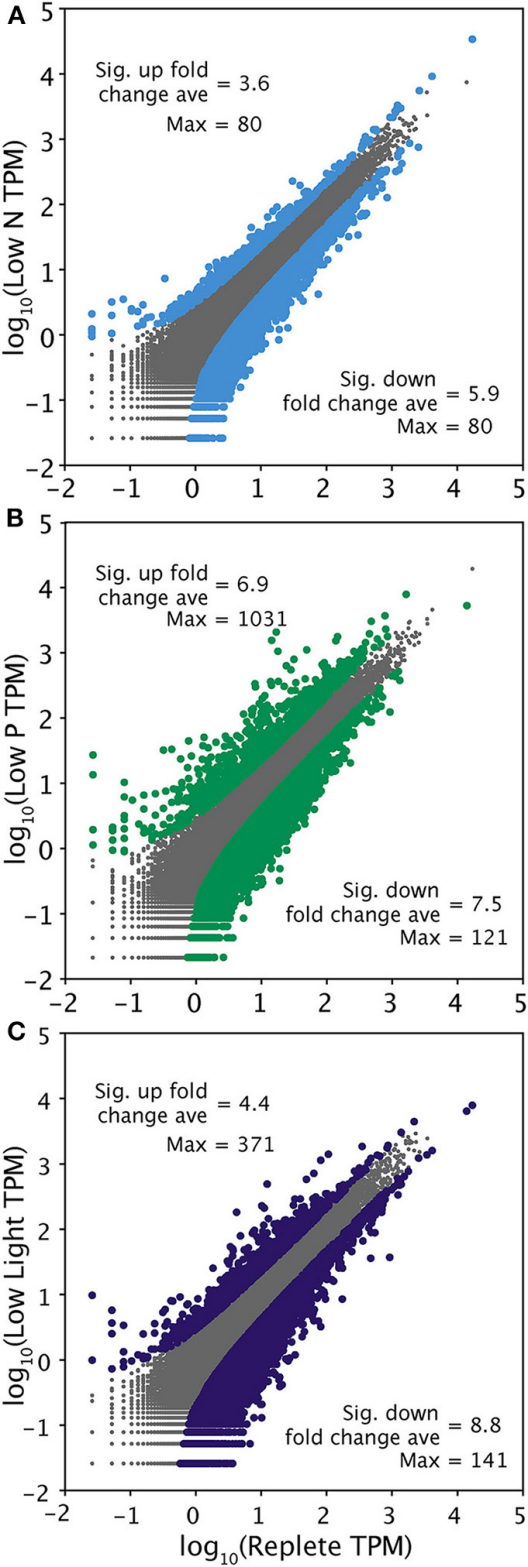
**Pairwise comparison of transcriptional responses across 37,314 contigs generated by a 90% clustering of the Trinity assembly in each treatment relative to the replete control**. Transcriptome-side gene expression patterns are shown in low N **(A)**, low P **(B)**, and low light **(C)**. Transcripts were normalized to total library size in tags per million (TPM). Gray points represent transcripts that were not significantly differentially regulated. Significance (colored points) was determined with ASC using a fold change greater than or equal to 2 and a posterior probability greater than 0.95 (Wu et al., 2010). The average significant fold change and maximum fold change value of up and downregulated transcripts is noted.

ASC was used to determine significant differential expression in the treatments relative to the replete condition control using the 90% clustered Trinity assembly (Wu et al., 2010). The low P condition elicited the strongest overall response, with more transcripts (1205) significantly upregulated than the other treatments and the largest fold changes (Figure 2; Table 4). The transcriptomic response to low N was the weakest overall, relative to the other treatments (Figure 2; Table 4). The Trinity and ABySS methods yielded similar numbers of differentially upregulated transcripts (Table 4). Although the genome mapping method recovered fewer significantly differentially regulated transcripts, this disparity is due to the difference in the number of gene models (11,501) vs. the number of contigs generated by the two *de novo* assemblies (>30,000). The percentage of differentially expressed transcripts relative to the total were comparable across all methods, with ~2.5% of all gene models or contigs significantly upregulated.

**Table 4:** **Significantly differentially upregulated transcripts from the mapping and assembly pipelines**.

	**Upregulated genes**			Low N^*^		**Low P**		**Low light**	
	**Low N**	**Low P**	**Low Light**	**vs. Trinity**	**vs. ABySS**	**vs. Trinity**	**vs. ABySS**	**vs. Trinity**	**vs. ABySS**
1984 gene models	91	298	182	88%	93%	94%	95%	93%	92%
Trinity	774	1205	998	–	89%	–	88%	–	76%
ABySS	762	1292	1228	57%	–	78%	–	57%	–

Significance was determined with ASC using a fold change greater than or equal to 2 and a posterior probability greater than 0.95 (Wu et al., 2010). Differences in the number of upregulated genes can be attributed the discrepancy between number of gene models and assembly size (Table 3); in all cases, ~2.5% of gene models/contigs were significantly differentially expressed.

* Comparisons between significant transcripts from each pipeline were tabulated by performing reciprocal BLAST alignments (Camacho et al., 2009) with e-value cutoffs of 1 × 10^−5^.

A careful examination of transcripts significantly upregulated under each condition was performed to identify biochemical pathways utilized by *A. anophagefferens* during the low N, P, and light conditions that prevail during brown tides. Significantly downregulated transcripts were not focused on as these largely represented genes indicative of the stationary phase of growth; as such, they were generally less informative of specific responses to the low nutrient or low light conditions tested. In all treatments, the Trinity and ABySS assemblies captured an average of 93% of the ASC-identified significantly differentially regulated gene models as determined by the genome mapping method (Table 4). Between 76 and 89% of significant Trinity contigs were homologous to ABySS contigs (Table 4). Furthermore, the functional annotations of the vast majority of significantly differentially expressed transcripts discussed in detail herein are present in the ASC significant sets across both *de novo* methods and the genome mapping method. The single exception to this is the transcript encoding the VTC4 polyphosphate polymerase. This contig aligns to the full *A. anophagefferens* genome but is not present in the published gene models, therefore this transcript was not detected by the genome mapping method. Overall, these results indicate that the biological interpretation presented here would be similar regardless of the assembly or mapping method utilized in downstream analyses. Additionally, these results show that *de novo* assemblies can enable the detection of important genes that are not included in the modeled gene set, as was the case for the VTC4 polyphosphate polymerase. In order to highlight the efficacy of *de novo* assembly methods in light of the constraints of the MMETSP, namely that the majority of datasets are generated from organisms without sequenced genomes, downstream analyses were performed on differential expression results from the Trinity *de novo* assembly, clustered at 90% sequence identity.

### Treatment specific responses

**Figure 3:**
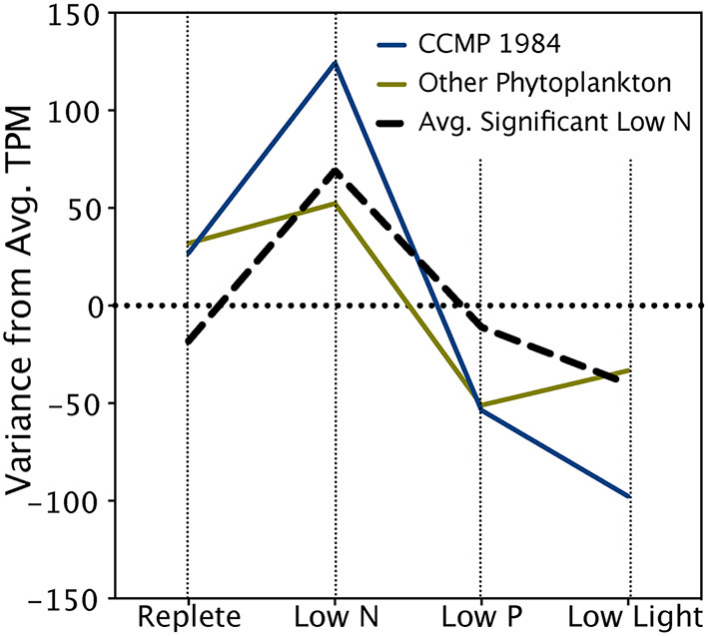
**Expression patterns of key nitrogen metabolism transcripts**. The black hashed line denotes the mean variance in expression across treatments of transcripts significantly upregulated in the low N treatment. Significance was determined with ASC using a fold change greater than or equal to 2 and a posterior probability greater than 0.95 (Wu et al., 2010). The blue line denotes the average variance in expression of transcripts from this study with functional annotations previously determined to be significantly upregulated during low N conditions in *A. anophagefferens* CCMP 1984 (Berg et al., 2008; Wurch et al., 2011b) (See Table 5). The green line denotes the variance in expression of transcripts from this study with functional annotations identified as upregulated during low N conditions in diatoms or coccolithophores (Dyhrman et al., 2006, 2012; Allen et al., 2011; Bender et al., 2014) (See Table 5).

The *A. anophagefferens* CCMP 1850 response to low N conditions was characterized by significantly upregulated transcripts encoding nitrate transporters, nitrate reductases and peptidases (Table 5). In addition to the transcripts that passed the stringent ASC significance cutoff, modeling of expression of N metabolism genes known to respond significantly to N limitation in *A. anophagefferens* CCMP 1984 (Berg et al., 2008; Wurch et al., 2011b) or other phytoplankton (Dyhrman et al., 2006; Allen et al., 2011; Bender et al., 2014) revealed that these transcripts mirror expression patterns in the significantly upregulated N-responsive set, with higher expression in the low N treatment relative to replete, low P and low light (Table 5; Figure 3).

**Table 5:** **The N-related transcripts discussed in this study**.

**Functional Annotation**	CCMP 1984 Up-regulated^**^	**Contig IDs**
Nitrite reductase^*^		**14256**
Nitrate transporter	X	**17069, 17070**
Peptidase	X	**30319, 30320, 26295, 28415, 26300**
Amino acid transporter^*^	X	29592, 10105, 13066
Ammonium transporter^*^	X	1328, 25931, 25928
Formamidase^*^	X	29974
Nitrite/formate transporter^*^	X	16166, 16167, 16168, 17069, 17070, 18408, 32854
Urea transporter^*^	X	14024
Xanthine/uracil/ vitaminC permease^*^	X	34780, 34781
Arginase^*^		417, 19577, 20151, 23473, 23474, 23476, 23478, 27885, 27887, 1681
Nitrate reductase^*^		12609, 23551
Urease^*^		9526

Bold contigs were deemed significantly differentially expressed using ASC (greater than 2-fold change relative to replete with a posterior probability of 0.95 or above; Wu et al., 2010).

* Denotes transcripts identified as N-regulated in other algae (Dyhrman et al., 2006, 2012; Berg et al., 2008; Allen et al., 2011; Wurch et al., 2011b; Bender et al., 2014) that follow a pattern of N regulation (Figure 3) in CCMP 1850.

** (Berg et al., 2008; Wurch et al., 2011b).

**Figure 4:**
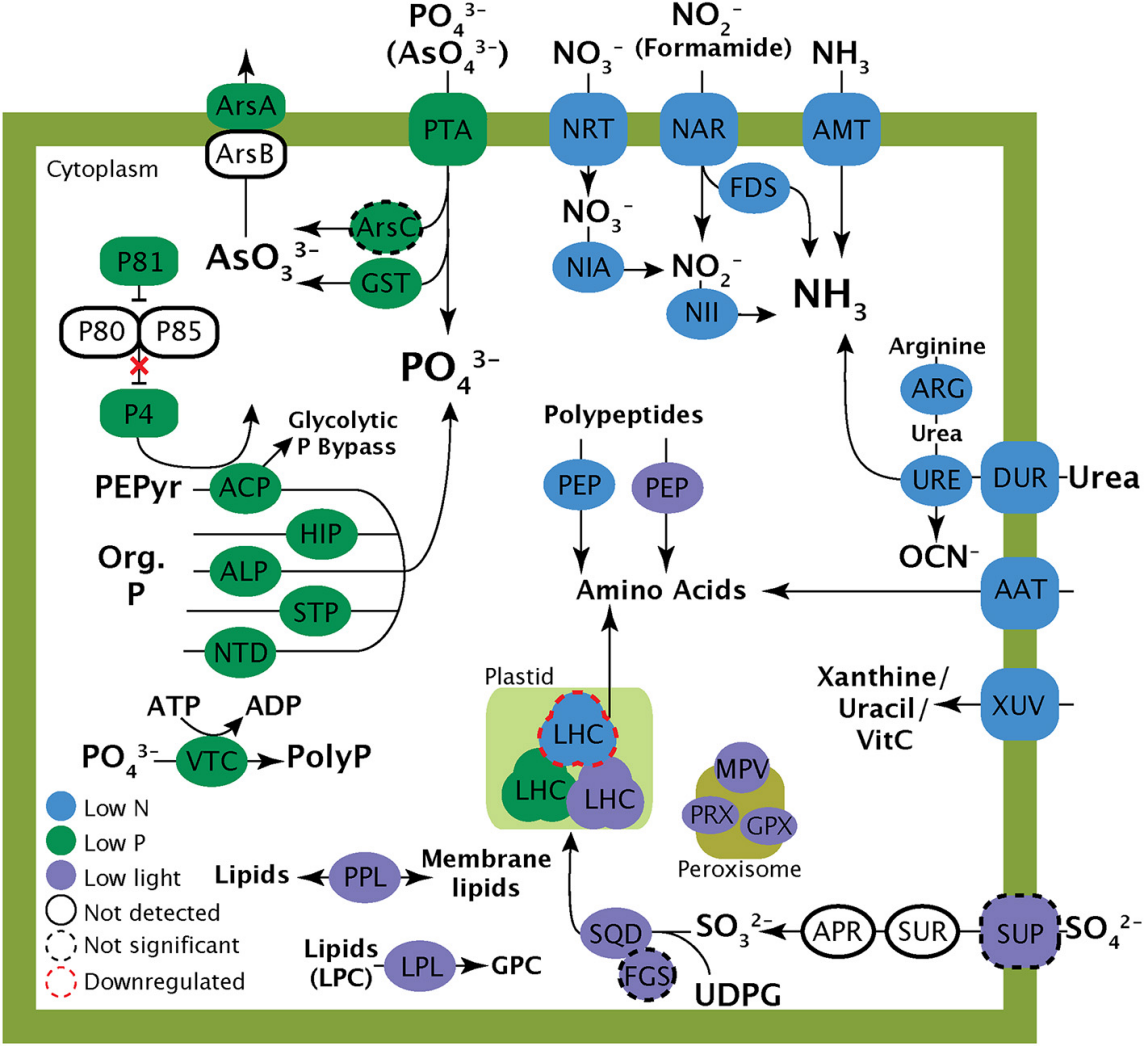
**Schematic cell model illustrating the potential role of the transcripts highlighted in this study**. Localization of the proteins depicted is for clarity and is not meant to represent actual protein localization in the cell. Proteins with black dashed lines represent transcripts that were detected in transcriptomes but were not significantly differentially expressed relative to the control. AAP, Amino acid permease; ACP, Acid phosphatase; ALP, Alkaline phosphatase; AMT, Ammonia transporter; APR, Adenosine-5′-phosphosulfate reductase, ARG, arginase; ArsA, Arsenite trasnlocating ATPase; ArsB, Arsenite efflux protein; ArsC, Arsenate reductase; DUR, Urea transporter; FDS, Formamidase; FGS, Ferredoxin-dependent glutamate synthase; GPX, Glutathione peroxidase; GST, Glutathione S Transferase; HIP, Histidine phosphatase; LHC, Light harvesting complex; LPL, Lysophospholipase; NAR, Nitrite transporter; NIA, Nitrate reductase; NII, Nitrite reductase; NRT, Nitrate transporter; NTD, 5′-Nucleotidase; P4-85, PHO pathway; PEP, Peptidase; PEPyr, Phosphoenolpyruvate; PPL, Patatin-like phospholipase; PTA, Phosphate transporter; SQD, SQD1 (sulfolipid biosynthesis gene); STP, Serine/threonine phosphatase; SUP, Sulfate permease; SUR, Sulfate reductase; UDPG, UDP-glucose; URE, Urease; VTC, Vacuolar transport chaperone (VTC4); XUV, Xanthine/uracil/Vitamin C permease.

The response to the low P treatment featured the significant upregulation of transcripts responsible for phosphate transport and the hydrolysis of DOP including several phosphatases and a 5′-nucleotidase (Figure 4; Table 6), concurrent with an increase in alkaline phosphatase activity. Transcripts for components of a PHO-like (Toh-e et al., 1981) P regulatory signaling cascade were found to be upregulated under low P (Figure 4; Table 6), as was a vacuolar transport chaperone 4 (VTC4) with homology to a eukaryotic polyphosphate polymerase (Hothorn et al., 2009) (Figure 4; Table 6). Transcripts encoding components of an arsenite detoxification pathway including an arsenite transporting ATPase and a glutathione S-transferase were also significantly upregulated (Figure 4; Table 6). An arsenate reductase was identified in the transcriptome, but was not significantly upregulated in the low P treatment relative to the replete control.

**Table 6:** **The P-related transcripts discussed in this study**.

**Functional annotation**	CCMP 1984 Upregulated^*^	**Contig IDs**
5′-nucleotidase	X	**30121, 30125, 10871, 10870**
Acid phosphatase		**10321**
Alkaline phosphatase	X	**10956**
Arsenite translocating ATPase	X	**30015, 30016, 11517, 5709, 4972, 34882, 20717**
Glutathione S-transferase		**32928, 34579, 35076**
Histidine phosphatase		**13355, 11210, 11211, 35179**
Inorganic phosphate trans.	X	**18415**
PHO4-like		**13492, 13491, 18417, 18419, 18421, 18418, 18415**
PHO81-like (SPX)		**26500, 20877**
Ser/Thr phosphatase		**16372, 7353, 16372, 611, 12799, 13773, 4312**
Vacuolar transport chaperone (VTC4)	X	**31531**

Bold contigs were deemed significantly differentially expressed using ASC (greater than 2-fold change relative to replete with a posterior probability of 0.95 or above; Wu et al., 2010).

* (Wurch et al., 2011b).

In *A. anophagefferens*, low light conditions resulted in the upregulation of genes involved in a variety of lipid modification and recycling reactions. Light stress uniquely resulted in the significant upregulation of transcripts encoding two different phospholipase-like enzymes: several identified as lysophospholipases and the other a patatin-like phospholipase (Figure 4; Table 7). A set of transcripts involved in the structure and activity of peroxisomes, including peroxidases/catalases, glutathione peroxidase, and an integral membrane protein of peroxisomes (MPV17) were also significantly upregulated in the low light treatment (Figure 4; Table 7). Finally, transcripts from the pathway for sulfolipid biosynthesis were detected in the low light treatment. A transcript encoding the sulfolipid biosynthesis protein SQD1 was significantly upregulated (Figure 4; Table 7). An accessory protein to the sulfolipid biosynthesis process, ferredoxin-dependent glutamate synthase (FdGOGAT) and a sulfate transporter were also detected in the low light transcriptome, but were not significantly upregulated relative to the replete treatment. A complete list of the patterns of abundance and differential expression statistics generated by ASC for each transcript are provided in the supplemental data (Data Sheet 1).

**Table 7:** **The light-related transcripts discussed in this study**.

**Functional annotation**	**Contig IDs**
Gutathione peroxidase	**35103, 24785**
Lysophospholipase	**12903, 21507, 12903**
Patatin-like phospholipase	**21507**
Peptidase	**34722, 13270, 33567, 34987, 27009, 21149, 16513, 4230**
Peroxidase/Catalase	**5250, 15891, 15889, 15898, 5250, 5251, 13780**
Peroxisome-membrane associated (MPV17)	**23809, 33103, 37161, 9842**
Sulfolipid biosynthesis protein (SQD1)	**4254**

Bold contigs were deemed significantly differentially expressed using ASC (greater than 2-fold change relative to replete with a posterior probability of 0.95 or above; Wu et al., 2010).

### Patterns in overlapping responses

**Figure 5:**
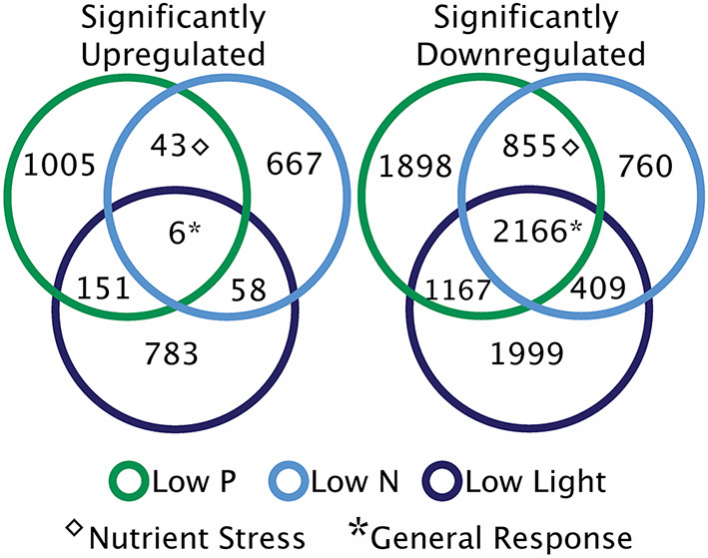
**Broad categories of significant transcriptional responses**. Diagrams illustrate the patterns of significantly differentially regulated genes across the treatments. Sections without overlap represent unique responses to each treatment. Overlapping sections are indicative of nutrient stress response transcripts [overlap between low nitrogen (N) and low phosphorus (P)], and shared low light and low N or P responses. The generally responsive category describes significantly up or downregulated transcripts shared across three treatments.

Response patterns are defined by identifying the overlap in significantly differentially regulated transcripts under the low nutrient and low light treatments (Figure 5). The overlap between significant transcripts from low N and low P conditions represents differential expression indicative of nutrient stress. The general response transcripts are defined here as those that are either upregulated or downregulated under all three treatments relative to the control (Figure 5). However, many of these general response transcripts could be jointly upregulated because they are responding to the switch to stationary phase and the cessation of exponential growth, rather than being a shared response to nutrient or light stress.

**Figure 6:**
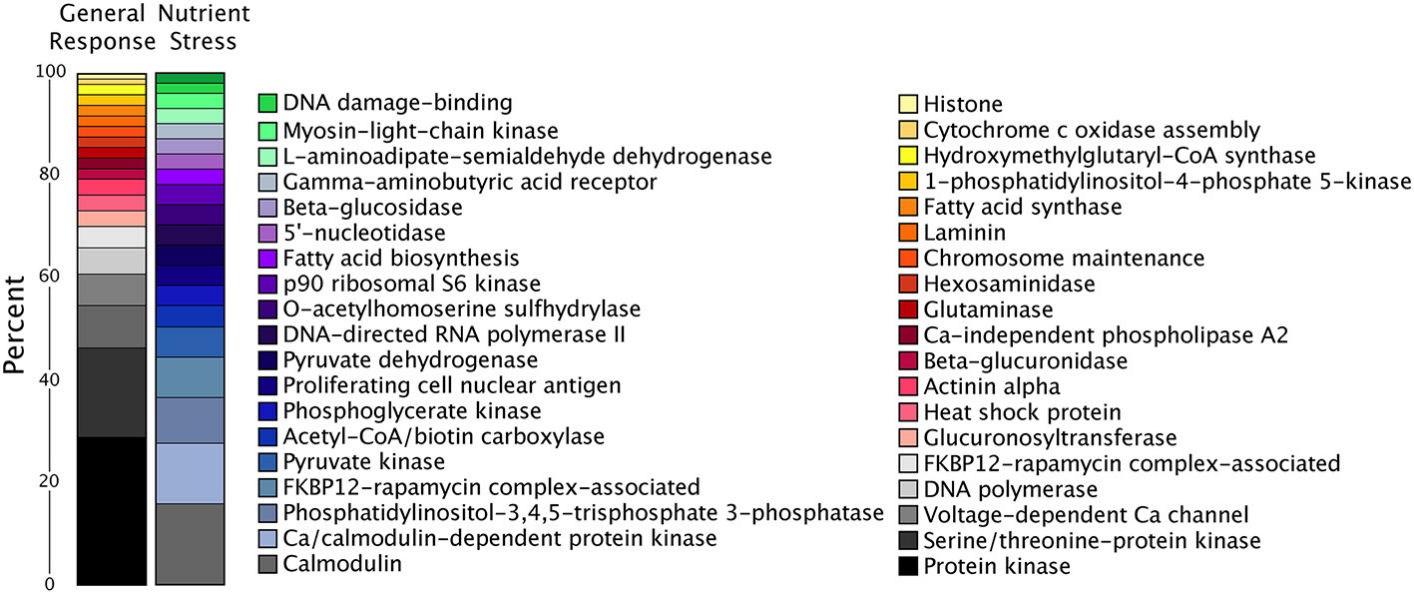
**Percentage of transcripts in the top 20 KEGG annotations for significantly downregulated transcripts in the general response and nutrient stress categories**. The only shared KEGG annotation between these two categories was calmodulin.

There were relatively very few transcripts upregulated in all three treatments or shared across two treatments, as compared to significantly downregulated transcripts overlapping between treatments (Figure 5). Two of the six shared upregulated transcripts encode for putative WLM domains and the other four transcripts share homology to an ADP-ribosylation factor. The majority of the 2166 shared downregulated transcripts encode protein kinases, DNA polymerase subunits, and proteins involved in signal transduction (such as calmodulin), falling into KEGG pathways like DNA replication, ribosome biogenesis, and purine and pyrimidine metabolism (Figure 6). During nutrient stress (low N and low P), the most numerous categories of KEGG classified downregulated transcripts include metabolic pathways, biosynthesis of secondary metabolites, purine synthesis and cell cycle regulation proteins (Figure 6). The most numerous general response downregulated transcripts in these KEGG categories were involved with signal transduction, protein phosphorylation, and transcripts involved in transcription or translation (Figure 6). The downregulation of the transcripts in these categories could also point to decreases in growth rate that accompany the onset of stationary phase when treatment cultures were harvested.

**Figure 7:**
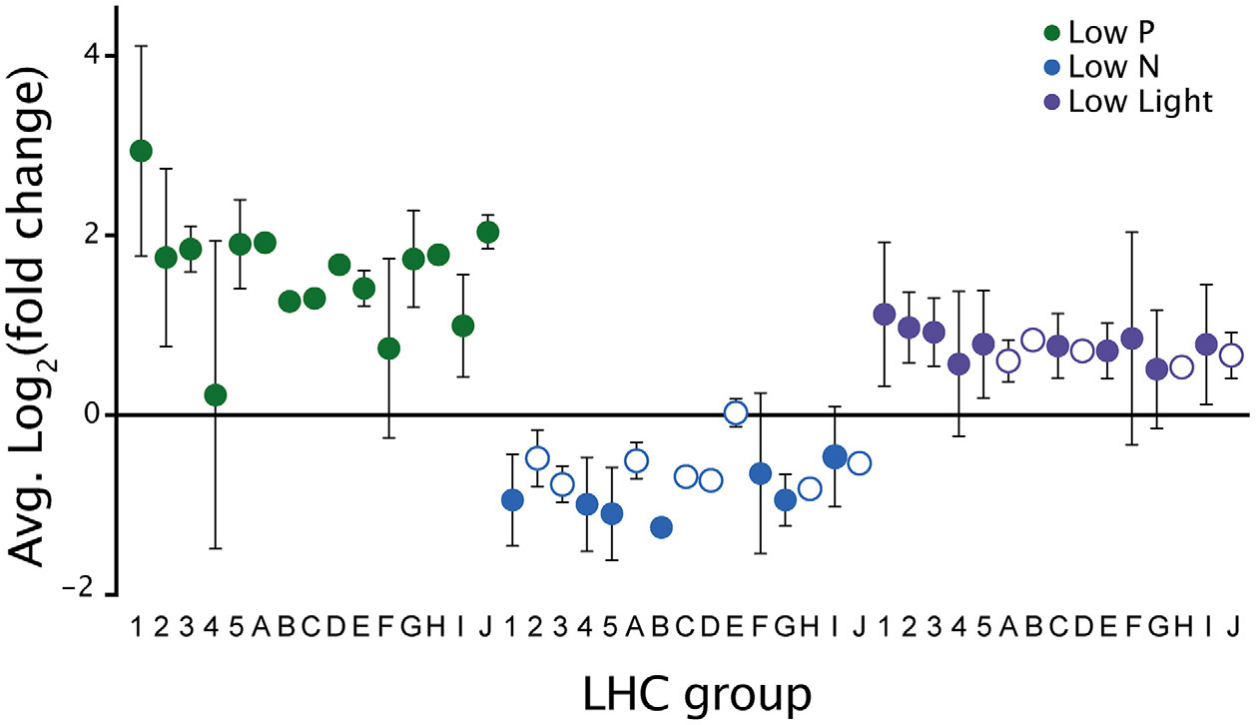
**Expression patterns of light harvesting complex (LHC) transcripts between treatments**. The notation on the x-axis refers to the phylogenetic groups of different LHC genes identified in the *A. anophagefferens* CCMP 1984 genome (Gobler et al., 2011). Filled circles indicate LHC groups with significantly differentially expressed contigs in the LHC group. Open circles indicate a lack of significant differentially expressed contigs in the LHC group. Error bars denote standard deviation.

Transcripts involved with photosynthetic processes were dynamically expressed across all treatments. There were different transcripts identified as encoding photosystem I and II proteins upregulated under all three treatments. In addition to these photosystem-encoding transcripts, a large number of significantly responsive transcripts in all treatments were identified as encoding light harvesting complex (LHC) proteins. CCMP 1984 genome analysis identified 62 LHC proteins, which fell into 16 distinct groups based on phylogenetic similarity to LHCs from other phytoplankton (Gobler et al., 2011). Significantly upregulated LHC contigs spanned 15 of the 16 LHC groups during low P and 10 groups during low light, while significantly downregulated contigs during low N spanned 7 groups (Figure 7). The downregulation of LHC transcripts is supported by the noted decrease in photosynthetic efficiency at the time of culture harvest in the low N sample (Table 1).

### Comparison to *A. anophagefferens* CCMP 1984

In the low N treatment, transcripts for nitrate transporters and peptidase were found to be significantly upregulated in both strains (Berg et al., 2008; Wurch et al., 2011b) (Table 5). Nitrite reductase was not observed to be significantly upregulated in low N in previous work on CCMP 1984, but was significantly upregulated in this study of CCMP 1850 (Table 5). The other significant N-responsive transcripts identified in CCMP 1984 included transporters of urea, ammonia, amino acids, nitrate, and xanthine/uracil/vitamin C, and formamidase (Berg et al., 2008; Wurch et al., 2011b) (Table 5). All of these genes were highly expressed in the low N treatment and followed an expression pattern similar to that of significantly upregulated N transcripts (Figure 3). Examination of low P transcripts in CCMP 1984 (Wurch et al., 2011b) identified considerable overlap with the CCMP 1850 low P transcriptome, including the upregulation of a 5′-nucleotidase, alkaline phosphatase, arsenite translocating ATPase, inorganic phosphate transporter, and a VTC4 polyphosphate polymerase (Table 6).

## Discussion

Full genome sequencing for eukaryotic phytoplankton is rare and with the recent exception of *E. huxleyi* (Read et al., 2013), genomes are only available for single isolates within a species (Palenik et al., 2007), and often only for a single species within the genus or even functional group (Armbrust et al., 2004; Gobler et al., 2011). The *de novo* assembly of high throughput transcript reads is an increasingly used and powerful alternative for the examination of strain differences, gene discovery, and expression analysis (Moustafa et al., 2010; Thompson et al., 2011; Dyhrman et al., 2012; Keeling et al., 2014). Here, high throughput sequencing was used to examine the toxic *A. anophagefferens* strain CCMP 1850 transcriptome, and how it was modulated under the low N, low P, and low light conditions that this alga may experience in an ecosystem setting during harmful algal blooms.

### Method comparisons

Comparison of the two *de novo* assemblies of strain CCMP 1850 to the CCMP 1984 genome found >95% of the assembled transcripts successfully aligned to the full CCMP 1984 genome scaffolds. Another indication of strain similarity was further observed, in that the majority of CCMP 1984 gene models were present in the two *de novo* assemblies with the Trinity pipeline performing somewhat better. Variability in gene model recovery could be related to sequence heterogeneity or variability in gene content between strains. It could also be that these genes are not expressed, or not expressed highly enough under the conditions used to build the assembly for them to be detected. A specific key difference between the genome strain CCMP 1984 and CCMP 1850 is the noted toxicity of the latter (Harke et al., 2011). No clear pathways for toxin production were detected by this study, most likely because the mechanism of toxicity of this alga is unclear (Gobler et al., 2005) and, hence, the genes responsible for mediating toxicity are likely novel. Little is known about the regulation of toxin production, but it is also possible that the transcripts for toxin production are not present in this assembly. Future conditional gene expression studies in toxic strains of *A. anophagefferens* would help elucidate pathways for toxin production.

The conditions used for the transcriptome assembly presented here provide an opportunity to examine differential responses in CCMP 1850 to the low inorganic nutrient and low light conditions that may prevail during blooms. Sequencing of biological replicates was not available as part of the MMETSP. To address this, samples in this study were pooled from triplicate cultures and then analyzed with a conservative statistical tool developed and optimized for analyzing non-replicated RNA-seq data. ASC is a Bayesian method which computes the posterior probability associated with different fold changes (Wu et al., 2010). ASC detects differentially expressed genes over a wide range of read counts and performs accurately but conservatively when compared to other methods on data with and without replicates (Wu et al., 2010). In short, while false discovery is low, the trade-off is that ASC generally recovers fewer significant genes compared to other bioinformatics methods applied with replicates (Wu et al., 2010). Hence, this approach may not detect some responses that are biologically relevant. This tool has been successfully implemented to study differential expression patterns in a number of peer-reviewed studies (Alexander et al., 2012; Dyhrman et al., 2012; Thomas et al., 2012; Konotchick et al., 2013).

Functional annotation of significantly expressed transcripts showed that pathways recovered were similar regardless of whether transcriptomes were assembled with *de novo* or genome mapping pipelines. An important exception to this is the VTC4 polyphosphate polymerase that was not among the CCMP 1984 gene models, but was present in the genome scaffolds. The detection of a significant and biologically important transcript within *de novo* assemblies that would not have been readily detected using genome mapping underscores the benefit of using these methods to facilitate gene discovery. In each transcriptome, roughly half of the significant transcripts remained unannotated after the annotation step; these transcripts could represent important conditional responses and future studies could focus on the determination of their function. While the functions of the discussed genes have not been experimentally validated, overall the results presented here underscore both the robust nature of *de novo* assemblies and the detection of consistent significant responses using ASC without replicates and with or without a genome. As such, other researchers performing similar analyses on MMETSP data may be able to confidently recover genes and assess differential responses regardless of the assembly method, the presence of a sequenced genome, or replicates.

### Low N responses in *A. anophagefferens* CCMP 1850

The low N treatment was characterized by the significant upregulation of transcripts with known roles in N metabolism including a nitrate transporter, nitrite reductase and a peptidase. This underscores that increased transport of inorganic N and utilization of DON sources are central responses to the low N conditions in which blooms occur. These responses emphasize the importance of DON metabolism in this species, which has been well-documented in other studies (Berg et al., 2008; Gobler et al., 2011; Wurch et al., 2011b).

Although the stringency of ASC limited the number of statistically significant responses with known roles in N metabolism detected in CCMP 1850, N-related transcriptional responses have been studied in some detail for other phytoplankton including diatoms and coccolithophores (Dyhrman et al., 2006; Allen et al., 2011; Bender et al., 2014). The homologs of N-responsive genes (e.g. nitrate transporter, urea transporter etc.) from other phytoplankton were present in the CCMP 1850 transcriptome, and their expression patterns closely paralleled those of the significant CCMP 1850 N-responsive transcripts. It is likely that these transcripts, which encode for arginase, nitrate reductase, and urease, among others, are biologically responding to the low exogenous inorganic N supply given their expression characteristics, even though the stringency of ASC prevented their inclusion among the pool of genes identified as significantly differentially expressed. Ultimately, given the patterns observed here, it is likely that the aforementioned transcripts are in fact important N responders and thus implicated in enabling *A. anophagefferens* success under the low N conditions that occur during blooms. In phytoplankton certain low N responses appear to be conserved. These include upregulation of nitrate transporters, which was observed in *E. huxleyi* and several diatoms (Dyhrman et al., 2006; Allen et al., 2011; Bender et al., 2014). Conversely, urea uptake and utilization pathways have more varied patterns of expression in response to low N, with upregulation of urea cycle transcripts observed in diatoms and *A. anophagefferens*, but not *E. huxleyi* (Dyhrman et al., 2006; Allen et al., 2011; Bender et al., 2014). The variability in expression of these pathways has implications for the influence of urea on niche segregation over other nutrients like nitrate in phytoplankton.

The observed downregulation of LHC-encoding transcripts in the low N treatment and the concurrent upregulation of protein recycling enzymes like peptidases hint at a mechanism of N sparing with the concomitant loss of photosynthetic efficiency. This is consistent with a marked decrease in chlorophyll *a* concentration and the observed decline in photosynthetic efficiency without a decrease in cell concentration at the time of harvesting the low N treatment. In other phytoplankton, environmental cues such as changes in light level and nutrient availability have been noted to alter the composition of LHC proteins (Grossman et al., 1993). Additionally, a transcriptional investigation of *E. huxleyi* noted a downregulation of LHC transcripts in response to low N (Dyhrman et al., 2006). Consistent with N-sparing through a reduction in N-rich proteins, transcripts encoding protein-degrading enzymes were significantly upregulated and could serve to recycle N in order to increase the pool of available N during times when exogenous concentrations are low.

### Low P responses in *A. anophagefferens* CCMP 1850

Transcriptional patterns in the low P treatment reflect the importance of phosphate transport, organic P utilization, P homeostasis, and the detoxification of arsenate. With the upregulation of a phosphate transporter and several transcripts encoding enzymes like 5′-nucleotidase and alkaline phosphatase, *A. anophagefferens* CCMP 1850 likely increases phosphate uptake and the hydrolysis of DOP under low P conditions. Notably, alkaline phosphatase activity was heightened in the low P treatment concurrent with the significant upregulation of the alkaline phosphatase transcript. These low P responses have also been detected in other phytoplankton including coccolithophores and diatoms at the transcriptional level (Dyhrman et al., 2006, 2012), and in many other physiological studies (Gonzalez-Gil et al., 1998; Dyhrman and Palenik, 1999; Chung et al., 2003; Dyhrman, 2005; Dyhrman and Ruttenberg, 2006) highlighting the conserved nature of this response. As with the patterns described above for DON, DOP likely plays a critical role in fueling the growth of this species during blooms.

Although these responses are typically conserved among eukaryotic algae, relatively little is known about the molecular-level signaling cascade that regulates these genes. In *Chlamydomonas* the P stress response is thought to be regulated in part by the induction of the transcription factor PSR1 that controls expression of phosphate transporters and phosphatases (Wykoff et al., 1999; Moseley et al., 2006). Although a possible homolog of *Chlamydomonas* PSR1 was detected in the CCMP 1850 transcriptome, it was not upregulated in response to low P. In yeast, there is a well-characterized cyclin-dependent PHO pathway (Lenburg and O'Shea, 1996), and the significant upregulation of a yeast PHO81 homolog in *A. anophagefferens* CCMP 1850 suggests that a yeast-like mechanism may be used to sense and respond to low P in *A. anophagefferens*. In *Aureococcus*, the relationship between P deprivation and bloom dynamics is increasingly recognized as important (Wurch et al., 2014) and the P sensor response system in this species warrants further study.

Potential P recycling and sparing strategies in *A. anophagefferens* CCMP 1850 are evident by the upregulation of an acid phosphatase and a polyphosphate polymerase. In higher plants, acid phosphatases are upregulated under low P (Veljanovski et al., 2006) where they act to circumvent the P-requiring steps of glycolysis by preferentially hydrolyzing phosphoenolpyruvate (PEP) (Lefebvre et al., 1990). Breakdown of PEP leads to a “glycolytic bypass” whereby carbon metabolism is able to progress while bypassing some P-requiring steps (Lefebvre et al., 1990). The presence of a glycolytic bypass has been hypothesized for diatoms (Dyhrman et al., 2012) and *A. anophagefferens* CCMP 1984 (Wurch et al., 2011a). It is also possible that the acid phosphatase is localized to an intracellular vacuole where it may be involved in polyphosphate cycling (Veljanovski et al., 2006). The modulation of polyphosphate pools is increasingly recognized as an important aspect of P homeostasis in low P conditions (Dyhrman et al., 2006, 2012; Martin and Van Mooy, 2013; Martin et al., 2014). The upregulation of the VTC4 polyphosphate polymerase (Hothorn et al., 2009), suggests that although luxury stores of polyphosphate may be mobilized during P stress, there may also be polyphosphate biosynthesis. This is consistent with observations of increased polyphosphate to total particulate phosphate ratios in phytoplankton from the low P Sargasso Sea (Martin et al., 2014). During low P conditions cells could experience a temporary excess of inorganic phosphate that could preemptively repress continued phosphate uptake. The upregulation of a polyphosphate polymerase during P stress conditions could enable the creation of a sink of readily accessible P while also circumventing repression of P scavenging (Ogawa et al., 2000). On the whole, the role of polyphosphate metabolism in P homeostasis is relatively unexplored in marine phytoplankton, and the presence of this response in *A. anophagefferens*, as well as coccolithophores and diatoms (Dyhrman et al., 2006, 2012) hints that induction of polyphosphate polymerases is an important and conserved P stress response.

In the low P transcriptome, an inorganic phosphate transporter was significantly upregulated; such transporters cannot typically discriminate between phosphate and arsenate and their upregulation could lead to an increase in intracellular arsenate (Budd and Craig, 1981; Silver and Phung, 2005). During low P conditions *A. anophagefferens* may mitigate arsenic toxicity by enzymatically reducing arsenate to arsenite and then pumping arsenite out of the cell. Eukaryotes have also been shown to employ an alternate method for arsenate reduction via a glutathione S-transferase (Zakharyan et al., 2005). This enzyme was significantly upregulated in the low P treatment, and it is possible that this represents a primary mode for arsenate reduction in *A. anophagefferens*. The resulting arsenite may be removed from the cell via an arsenite translocating ATPase, which was upregulated in the low P treatment. Some eukaryotic proteins with homology to arsenite translocating ATPases are implicated in gas vesicle formation and carbon starvation responses rather than arsenate detoxification (Castillo and Saier, 2010), but it is common for these pathways to be induced during low P in cyanobacteria (Cervantes et al., 1994; Rahman and Hassler, 2014). No transcripts encoding proteins in metal detoxification pathways were upregulated in the low P treatments of *T. pseudonana* (Dyhrman et al., 2012) or *E. huxleyi* (Dyhrman et al., 2006). This indicates that a highly responsive arsenic detoxification pathway could be a uniquely important physiological strategy for *A. anophagefferens* to bloom in coastal systems with high metal concentrations. Consistent with this idea, the greater abundance of metalloenzymes in *A. anophagefferens* compared to other phytoplankton has previously been interpreted as a sign of genomic adaptation to high metal concentrations in coastal environments (Gobler et al., 2011).

The uptake of arsenate could be amplified in coastal environments where arsenate concentrations are relatively high (Sanders, 1985) and ratios of As:P could be increased during the low inorganic P conditions that accompany brown tide blooms (Wurch et al., 2014). In the surface waters of oxidizing marine systems, arsenate is the most abundant inorganic arsenic compound (Sanders and Windom, 1980). In marine systems, microbial activities have been shown to modulate arsenic geochemistry and it has been noted that arsenite is the dominant species in systems with low P (Cutter and Cutter, 2006), suggestive of microbial detoxification. In *A. anophagefferens*, arsenate uptake, detoxification through arsenite efflux, and its potential role in altering coastal geochemistry have yet to be studied. Given the low and sometimes growth limiting inorganic P concentrations that have been observed during brown tide bloom events (Gobler et al., 2004; Wurch et al., 2014), the role of arsenic in *A. anophagefferens* eco-physiology is an important area of future research.

### Low light response in *A. anophagefferens* CCMP 1850

In *A. anophagefferens*, growth under low light conditions resulted in the upregulation of transcripts related to lipid metabolism and cycling, the breakdown of organic molecules, and restructuring of the photosynthetic apparatus. The upregulated transcripts encoding lipid-specific enzymes like lysophospholipases have not been characterized in eukaryotic phytoplankton, but in bacteria they are cell membrane localized and are the first step in lipid scavenging for membrane incorporation (Pride et al., 2013). Patatin-like phospholipases are widely distributed in prokaryotes and eukaryotes and are implicated in an array of functions including triacylglyceride metabolism, lipid membrane recycling, and membrane trafficking (Wilson, 2006).

Peroxisome activity could be another important lipid-related, low light response to organic matter processing. Transcripts identified as encoding integral peroxisome membrane components, peroxidase/catalase and glutathione peroxidase were significantly upregulated in the low light treatment. Antioxidant enzymes have been shown to respond to stress conditions in other phytoplankton (Pinto et al., 2003). There are many different specific functions of peroxisomes, however many contain antioxidant enzymes and are implicated in the oxidation of long chain fatty acids (Stabenau et al., 1989). Whether exogenous lipids were being catabolized as an energy source or recycled back into membranes to avoid the high energetic cost of fatty acid biosynthesis is unknown. Uptake and metabolism of long chain fatty acids is rare in bacteria (Pride et al., 2013), largely uncharacterized in phytoplankton, and has been previously overlooked as a mechanism that could allow *A. anophagefferens* to thrive under the low light conditions that are pervasive during dense brown tides.

The oxidation of organic molecules could help to explain the ability of *A. anophagefferens* to survive in conditions generally unfavorable for phototrophic growth. During experimental prolonged dark incubation, the cellular chlorophyll *a* content of *A. anophagefferens* did not decline, despite slight decreases in total cellular carbohydrates, proteins, and lipids (Popels et al., 2007). The pattern of utilization of cellular reserves appears to be conserved across eukaryotic phytoplankton, starting preferentially with the breakdown of starch, followed by proteolytic recycling of peptides, and ultimately the utilization of lipids (Handa, 1969; Griffiths, 1973; Dehning and Tilzer, 1989). The low light transcriptional responses identified here support the occurrence of these processes in *A. anophagefferens* during low light conditions.

The significant expression of transcripts involved in sulfolipid biosynthesis was a unique response to low light treatment. Despite their wide occurrence, sulfolipids are not essential for growth in autotrophs and their biosynthesis and substitution for phospholipids has been shown to serve mainly as a P stress response (Van Mooy et al., 2009; Martin et al., 2011; Wurch et al., 2011a). In *A. anophagefferens*, the concurrent upregulation of transcripts for sulfolipid biosynthesis and transcripts encoding photosystem proteins like LHCs could reflect an attempt to increase plastid membrane surface area to maximize photosynthetic yield during low light levels. This is corroborated by the expression of lipid recycling enzymes in the low P and low light treatments.

It is well-established that in low light photosynthetic cells increase pigment concentration and plastid membrane production (Archer et al., 1997). In the environment, dense brown tide blooms are accompanied by attenuated light conditions. Thus, low light could result in an altered P demand depending on how much phospholipid is recycled, replaced with sulfolipids, or newly synthesized as membrane area increases to accommodate more pigments. If there is an increase in phospholipid biosynthesis associated with low light, then *A. anophagefferens* blooms could experience enhanced P demand and subsequent P stress under the low light levels that accompany elevated cell densities. Consistent with this hypothesis, recent fieldwork has identified P stress responses in high density populations of *A. anophagefferens* (Wurch et al., 2014) and former work has found P limitation of *A. anophagefferens* net growth rates during blooms (Gobler et al., 2004). It has been established in ecosystem (Van Mooy et al., 2009) and laboratory studies (Martin et al., 2011; Wurch et al., 2011a; Dyhrman et al., 2012) that phytoplankton (including *A. anophagefferens* CCMP 1984) switch phospholipids for sulfolipids as a P conservation strategy. The results of this study indicate that the expression of sulfolipid biosynthesis genes might not be specific enough to assess P stress from light stress in field populations. Instead, the lipid composition of phytoplankton cells might be more diagnostic, as cells compensating for low P concentrations would have a higher ratio of sulfolipid to phospholipid relative to cells in replete conditions, whereas cells in low light would more likely have similar ratios but higher overall amounts of both lipids.

### Overlapping transcriptional responses

There were few transcripts that were jointly upregulated across all treatments. This could indicate that during stress conditions the transcriptional response of *A. anophagefferens* is uniquely tailored to the environment. The lack of overlap also illustrates the potential to use significant condition-responsive transcripts as biomarkers of environmental conditions in the field. In contrast to the low number of general stress and nutrient stress transcripts that were upregulated, these overlap categories had high number of transcripts downregulated relative to the control. Based on their KEGG identification, a high number these transcripts could be related to cell division or growth processes and are likely indicative of the stationary phase that these treatments were entering at the time of harvest.

During nutrient stress, a high proportion of genes for the synthesis of secondary metabolites were downregulated. It has been noted that at the height of brown tide blooms, *A. anophagefferens* is only minimally subjected to grazing (Gobler et al., 2005; Sunda et al., 2006). The production of secondary metabolites could be an important facet of grazer deterrence and the genome of *A. anophagefferens* has been shown to contain significantly more genes involved in the biosynthesis of these metabolites than other phytoplankton (Gobler et al., 2011). It is possible that slowed production of secondary metabolites when nutrient supplies diminish may leave this species more vulnerable to predation pressure that may contribute to the demise of brown tides.

### Strain responses to low N and low P

Previous low-resolution global transcriptional studies of *A. anophagefferens* CCMP 1984 (Wurch et al., 2011b) and targeted work on a suite of N metabolism genes (Berg et al., 2008) enabled the assessment of potential strain heterogeneity in response to low N and low P for this species. However, these comparisons carry several caveats. Subtle differences in the time of cell harvest and the growth conditions could impact the observed signals dramatically. Further, the CCMP 1984 Long-SAGE study by Wurch et al. (2011b) only examined the most abundant transcripts with NlaIII restriction sites. Despite these caveats there was overlap of significant nutrient responsive genes between the strains.

In both strains, the low P response was characterized by significantly upregulating transcripts for phosphate transport, DOP hydrolysis, the modulation of intracellular polyphosphate, and the detoxification of arsenic. Notably, the regulation of the phosphate transporter has been shown to be tightly regulated by P in CCMP 1984, and is expressed in field populations (Wurch et al., 2014). Its joint presence in both strains suggests both the consistency of the strain responses, and its potential utility as a biomarker of P stress in field populations.

There was also considerable strain overlap in response to low N. Shared significant functional responses included increased nitrate transport and peptidase activity (Berg et al., 2008; Wurch et al., 2011b), which again emphasizes the importance of DON metabolism in this species. By querying the CCMP 1850 transcripts for genes with known N regulation patterns in other algae and CCMP 1984, a number of putatively N-responsive transcripts were identified that followed an N-responsive pattern but were not detected as significant with our conservative application of ASC. These transcripts, putatively N-regulated in both strains, were found to encode a suite of transporters and enzymes for the transport and metabolism of DON, such as a xanthine/uracil/vitamin C permease, a formamidase, and an amino acid transporter among others. The xanthine/uracil/vitaminC permease is tightly regulated by N in CCMP 1984 and expressed in field populations (Wurch et al., 2014). More detailed expression work in CCMP 1850 may confirm its utility as a biomarker of N stress, although with the data herein, the nitrate transporter may be a better candidate given its significant response in both strains (Berg et al., 2008; Wurch et al., 2011b). Overall, the overlapping responses to nutrient deficiency reinforce the importance of traits that are critical to this HAB-forming organism's ecological success: the ability to utilize or recycle a wide range of N and P forms, especially DON and DOP.

## Conclusions

Assembly of 50 bp reads generated from high throughput sequence data from *A. anophagefferens* CCMP 1850 and differential expression analysis using ASC (Wu et al., 2010) revealed that genome mapping and *de novo* assembly pipelines can yield similar results, thus highlighting the efficacy of these approaches for the analysis of future MMETSP datasets. These findings underscore the utility of transcriptome profiling for gene discovery, examining strain differences and differential expression analysis, even in the absence of a sequenced genome. Taken in whole, the transcriptional responses to the test conditions in CCMP 1850 and the presence of similar responses in strain CCMP 1984 underscore that the ability to utilize and recycle organic compounds is a critical, niche-defining stress response in *A. anophagefferens.* Previous studies have assessed the range of strain level variation within a single phytoplankton species by examining the metabolic potential encoded in the genome (Read et al., 2013). The results presented here underscore that strain differentiation can also be considered at the transcriptional level, as differentiation has the potential to be conditional and dynamic, rather than inherent and static. Future comparison of transcriptional responses across conditions and between strains of *A. anophagefferens* will be a useful tool in the exploration of the eco-physiology of this organism.

### Conflict of interest statement

The authors declare that the research was conducted in the absence of any commercial or financial relationships that could be construed as a potential conflict of interest.

## References

[B1] Alexander H. Jenkins B. D. Rynearson T. A. Saito M. A. Mercier M. L. Dyhrman S. T. (2012). Identifying reference genes with stable expression from high throughput sequence data. Front. Microbiol. 3:385 10.3389/fmicb.2012.00385 23162540 PMC3494082

[B2] Allen A. E. Dupont C. L. Oborník M. Horák A. Nunes-Nesi A. McCrow J. P. (2011). Evolution and metabolic significance of the urea cycle in photosynthetic diatoms. Nature 473, 203–207 10.1038/nature10074 21562560

[B3] Altschul S. F. Gish W. Miller W. Myers E. W. Lipman D. J. (1990). Basic local alignment search tool. J. Mol. Biol. 215, 403–410 2231712 10.1016/S0022-2836(05)80360-2

[B4] Anderson D. M. Glibert P. M. Burkholder J. M. (2002). Harmful algal blooms and eutrophication: nutrient sources, composition, and consequences. Estuaries 25, 704–726 10.1007/BF02804901

[B5] Archer S. D. McDonald K. A. Jackman A. P. (1997). Effect of light irradiance on the production of sulfolipids from *Anabaena* 7120 in a fed-batch photobioreactor. Appl. Biochem. Biotechnol. 67, 139–152 9382487 10.1007/BF02787848

[B6] Armbrust E. V. Berges J. A. Bowler C. Green B. R. Martinez D. Putnam N. H. (2004). The genome of the diatom *Thalassiosira pseudonana*: ecology, evolution, and metabolism. Science 306, 79–86 10.1126/science.1101156 15459382

[B7] Bateman A. (2004). The Pfam protein families database. Nucleic Acids Res. 32, 138D–141D 10.1093/nar/gkh121 14681378 PMC308855

[B8] Bender S. J. Durkin C. A. Berthiaume C. T. Morales R. L. Armbrust E. V. (2014). Transcriptional responses of three model diatoms to nitrate limitation of growth. Front.Mar. Sci. 1:3 10.3389/fmars.2014.00003

[B9] Berg G. M. Shrager J. Glöckner G. Arrigo K. R. Grossman A. R. (2008). Understanding nitrogen limitation in *Aureococcus anophagefferens* (Pelagophyceae) through cDNA and qRT-PCR analysis. J. Phycol. 44, 1235–1249 10.1111/j.1529-8817.2008.00571.x 27041720

[B10] Birol I. Jackman S. D. Nielsen C. B. Qian J. Q. Varhol R. Stazyk G. (2009). *De novo* transcriptome assembly with ABySS. Bioinformatics 25, 2872–2877 10.1093/bioinformatics/btp367 19528083

[B11] Boneillo G. E. Mulholland M. R. (2013). Interannual variability influences brown tide (*Aureococcus anophagefferens*) blooms in coastal embayments. Estuaries Coasts 37, 1–17 10.1007/s12237-013-9683-3

[B12] Budd K. Craig S. R. (1981). Resistance to arsenate toxicity in the blue-green alga *Synechococcus leopoliensis*. Botany 59, 1518–1521

[B13] Camacho C. Coulouris G. Avagyan V. Ma N. Papadopoulos J. Bealer K. (2009). BLAST+: architecture and applications. BMC Bioinformatics 10:421 10.1186/1471-2105-10-421 20003500 PMC2803857

[B14] Castillo R. Saier M. H. (2010). Functional promiscuity of homologues of the bacterial ArsA ATPases. Int. J. Microbiol. 2010, 1–21 10.1155/2010/187373 20981284 PMC2963123

[B15] Cervantes C. Ramirez J. Silver S. (1994). Resistance to arsenic compounds in microorganisms. FEMS Microbiol. Rev. 15, 355–367 7848659 10.1111/j.1574-6976.1994.tb00145.x

[B16] Chung C. Hwang S. L. Chang J. Chung C. Hwang S. L. Chang J. (2003). Identification of a high-affinity phosphate transporter gene in a Prasinophyte alga, *Tetraselmis chui*, and its expression under nutrient limitation (2003). Appl. Environ. Microbiol. 69, 754–759 10.1128/AEM.69.2.754-759.2003 12570992 PMC143611

[B17] Cutter G. A. Cutter L. S. (2006). Biogeochemistry of arsenic and antimony in the North Pacific Ocean. Geochem. Geophys. Geosyst. 7, 1–12 10.1029/2005GC001159

[B18] Dehning I. Tilzer M. M. (1989). Survival of *Scenedesmus acuminatus* (Chlorophyceae) in darkness. J. Phycol. 25, 509–515

[B19] Doblin M. A. Blackburn S. I. Hallegraeff G. M. (1999). Growth and biomass stimulation of the toxic dinoflagellate *Gymnodinium catenatum* (Graham) by dissolved organic substances. J. Exp. Mar. Biol. Ecol. 236, 33–47

[B21] Dyhrman S. (2005). Ectoenzymes in *Prorocentrum minimum*. Harmful Algae 4, 619–627 10.1016/j.hal.2004.08.011

[B22] Dyhrman S. T. Haley S. T. Birkeland S. R. Wurch L. L. Cipriano M. J. McArthur A. G. (2006). Long serial analysis of gene expression for gene discovery and transcriptome profiling in the widespread marine coccolithophore *Emiliania huxleyi*. Appl. Env. Microbiol. 72, 252–260 10.1128/AEM.72.1.252-260.2006 16391051 PMC1352234

[B24] Dyhrman S. T. Jenkins B. D. Rynearson T. A. Saito M. A. Mercier M. L. Alexander H. (2012). The transcriptome and proteome of the diatom *Thalassiosira pseudonana* reveal a diverse phosphorus stress response. PLoS ONE 7:e33768 10.1371/journal.pone.0033768 22479440 PMC3315573

[B20] Dyhrman S. T. Palenik B. (1999). Phosphate stress in cultures and field populations of the dinoflagellate *Prorocentrum minimum* detected by a single-cell alkaline phosphatase assay. Appl. Env. Microbiol. 65, 3205–3212 10388722 10.1128/aem.65.7.3205-3212.1999 PMC91475

[B23] Dyhrman S. T. Ruttenberg K. C. (2006). Presence and regulation of alkaline phosphatase activity in eukaryotic phytoplankton from the coastal ocean: Implications for dissolved organic phosphorus remineralization. Limnol. Oceangr. 51, 1381–1390 10.4319/lo.2006.51.3.1381

[B25] Fu L. Niu B. Zhu Z. Wu S. Li W. (2012). CD-HIT: accelerated for clustering the next-generation sequencing data. Bioinformatics 28, 3150–3152 10.1093/bioinformatics/bts565 23060610 PMC3516142

[B26] Gobler C. J. Berry D. L. Dyhrman S. T. Wilhelm S. W. Salamov A. Lobanov A. V. (2011). Niche of harmful alga *Aureococcus anophagefferens* revealed through ecogenomics. Proc. Natl. Acad. Sci. U.S.A. 108, 4352–4357 10.1073/pnas.1016106108 21368207 PMC3060233

[B27] Gobler C. J. Boneillo G. E. Debenham C. Caron D. A. (2004). Nutrient limitation, organic matter cycling, and plankton dynamics during an *Aureococcus anophagefferens* bloom. Aquatic Microbial Ecol. 35, 31–43 10.3354/ame035031

[B28] Gobler C. J. Lonsdale D. J. Boyer G. L. (2005). A review of the causes, effects, and potential management of harmful brown tide blooms caused by *Aureococcus anophagefferens* (Hargraves et sieburth). Estuaries 28, 726–749 10.1007/BF02732911

[B29] Gobler C. J. Sunda W. G. (2012). Ecosystem disruptive algal blooms of the brown tide species, *Aureococcus anophagefferens* and *Aureoumbra lagunensis*. Harmful Algae 14, 36–45 10.1016/j.hal.2011.10.013

[B30] Gonzalez-Gil S. Keafer B. A. Jovine R. V. M. Aguilera A. Lu S. Anderson D. M. (1998). Detection and quantification of alkaline phosphatase in single cells of phosphorus-starved marine phytoplankton. Mar. Ecol. Prog. Ser. 164, 21–35

[B31] Gough J. Karplus K. Hughey R. Chothia C. (2001). Assignment of homology to genome sequences using a library of hidden Markov models that represent all proteins of known structure. J. Mol. Biol. 313, 903–919 10.1006/jmbi.2001.5080 11697912

[B32] Grabherr M. G. Haas B. J. Yassour M. Levin J. Z. Thompson D. A. Amit I. (2011). Full-length transcriptome assembly from RNA-Seq data without a reference genome. Nat. Biotechnol. 29, 644–652 10.1038/nbt.1883 21572440 PMC3571712

[B33] Griffiths D. J. (1973). Factors affecting the photosynthetic capacity of laboratory cultures of the diatom *Phaeodactylum tricornutum*. Mar. Biol. 21, 91–97

[B34] Grossman A. R. Schaefer M. R. Chiang G. G. Collier J. L. (1993). The phycobilisome, a light-harvesting complex responsive to environmental conditions. Microbiol. Rev. 57, 725–749 8246846 10.1128/mr.57.3.725-749.1993 PMC372933

[B35] Haft D. H. Loftus B. J. Richardson D. L. Yang F. Eisen J. A. Paulsen I. T. (2001). TIGRFAMs: a protein family resource for the functional identification of proteins. Nucleic Acids Res. 29, 41–43 10.1093/nar/29.1.41 11125044 PMC29844

[B36] Handa N. (1969). Carbohydrate metabolism in the marine diatom *Skeletonema costatum*. Mar. Biol. 4, 208–214

[B37] Harke M. J. Gobler C. J. Shumway S. E. (2011). Suspension feeding by the Atlantic slipper limpet (*Crepidula fornicata*) and the northern quahog (*Mercenaria mercenaria*) in the presence of cultured and wild populations of the harmful brown tide alga, *Aureococcus anophagefferens.* Harmful Algae 10, 503–511 10.1016/j.hal.2011.03.005

[B38] Heisler J. Glibert P. M. Burkholder J. M. Anderson D. M. Cochlan W. Dennison W. C. (2008). Eutrophication and harmful algal blooms: a scientific consensus. Harmful Algae 8, 3–13 10.1016/j.hal.2008.08.006 PMC5543702 28781587

[B39] Holm L. Sander C. (1998). Removing near-neighbour redundancy from large protein sequence collections. Bioinformatics 14, 423–429 9682055 10.1093/bioinformatics/14.5.423

[B40] Hoppe H. G. (1983). Significance of exoenzymatic activities in the ecology of brackish water: measurements by means of methylumbelliferyl-substrates. Mar. Ecol. Prog. Ser. 11, 299–308

[B41] Hothorn M. Neumann H. Lenherr E. D. Wehner M. Rybin V. Hassa P. O. (2009). Catalytic core of a membrane-associated eukaryotic polyphosphate polymerase. Science 324, 513–516 10.1126/science.1168120 19390046

[B42] Huang X. (1999). CAP3: a DNA sequence assembly program. Genome Res. 9, 868–877 10508846 10.1101/gr.9.9.868 PMC310812

[B43] Iseli C. Jongeneel C. V. Bucher P. (1999). ESTScan: a program for detecting, evaluating, and reconstructing potential coding regions in EST sequences. Proc. Int. Conf. Intell. Syst. Mol. Biol. 99, 138–148 10786296

[B44] Jones M. N. (1984). Nitrate reduction by shaking with cadmium: alternative to cadmium columns. Water Res. 18, 643–646

[B45] Kana T. M. Lomas M. W. MacIntyre H. L. Cornwell J. C. Gobler C. J. (2004). Stimulation of the brown tide organism, *Aureococcus anophagefferens*, by selective nutrient additions to *in situ* mesocosms. Harmful Algae 3, 377–388 10.1016/j.hal.2004.06.008

[B46] Kanehisa M. (2006). From genomics to chemical genomics: new developments in KEGG. Nucleic Acids Res. 34, D354–D357 10.1093/nar/gkj102 16381885 PMC1347464

[B47] Keeling P. J. Burki F. Wilcox H. M. Allam V. Allen E. E. Amaral-Zettler L. A. (2014). The marine microbial eukaryote transcriptome sequencing project (MMETSP): illuminating the functional diversity of eukaryotic life in the oceans through transcriptome sequencing. PLoS Biol. 12:e1001889 10.1371/journal.pbio.1001889 24959919 PMC4068987

[B48] Konotchick T. Dupont C. L. Valas R. E. Badger J. H. Allen A. E. (2013). Transcriptomic analysis of metabolic function in the giant kelp, *Macrocystis pyrifera*, across depth and season. New Phytol. 198, 398–407 10.1111/nph.12160 23488966 PMC3644879

[B49] Langmead B. Salzberg S. L. (2012). Fast gapped-read alignment with Bowtie 2. Nature 9, 357–359 10.1038/nmeth.1923 22388286 PMC3322381

[B50] Lefebvre D. D. Duff S. M. Fife C. A. Julien-Inalsingh C. Plaxton W. C. (1990). Response to phosphate deprivation in *Brassica nigra* suspension cells : enhancement of intracellular, cell surface, and secreted phosphatase activities compared to increases in pi-absorption rate. Plant Physiol. 93, 504–511 16667495 10.1104/pp.93.2.504 PMC1062541

[B51] Lenburg M. E. O'Shea E. K. (1996). Signaling phosphate starvation. Trends Biochem. Sci. 21, 383–387 8918192

[B52] Li R. Li Y. Kristiansen K. Wang J. (2008). SOAP: short oligonucleotide alignment program. Bioinformatics 24, 713–714 10.1093/bioinformatics/btn025 18227114

[B53] Li W. Godzik A. (2006). Cd-hit: a fast program for clustering and comparing large sets of protein or nucleotide sequences. Bioinformatics 22, 1658–1659 10.1093/bioinformatics/btl158 16731699

[B54] Lindner R. Friedel C. C. (2012). A comprehensive evaluation of alignment algorithms in the context of RNA-seq. PLoS ONE 7:e52403 10.1371/journal.pone.0052403 23300661 PMC3530550

[B55] Lohse M. Bolger A. M. Nagel A. Fernie A. R. Lunn J. E. Stitt M. (2012). RobiNA: a user-friendly, integrated software solution for RNA-Seq-based transcriptomics. Nucleic Acids Res. 40, W622–W627 10.1093/nar/gks540 22684630 PMC3394330

[B56] Lottaz C. Iseli C. Jongeneel C. V. Bucher P. (2003). Modeling sequencing errors by combining Hidden Markov models. Bioinformatics 19, ii103–ii112 10.1093/bioinformatics/btg1067 14534179

[B59] Martin P. Dyhrman S. T. Lomas M. W. Poulton N. J. Van Mooy B. A. S. (2014). Accumulation and enhanced cycling of polyphosphate by Sargasso Sea plankton in response to low phosphorus. Proc. Natl. Acad. Sci. U.S.A. 111, 8089–8094 10.1073/pnas.1321719111 24753593 PMC4050623

[B57] Martin P. Van Mooy B. A. S. (2013). Fluorometric quantification of polyphosphate in environmental plankton samples: extraction protocols, matrix effects, and nucleic acid interference. Appl. Environ. Microbiol. 79, 273–281 10.1128/AEM.02592-12 23104409 PMC3536087

[B58] Martin P. Van Mooy B. A. S. Heithoff A. Dyhrman S. T. (2011). Phosphorus supply drives rapid turnover of membrane phospholipids in the diatom *Thalassiosira pseudonana*. ISME J. 5, 1057–1060 10.1038/ismej.2010.192 21160536 PMC3131859

[B60] Moseley J. L. Chang C. Arthur G. (2006). Genome-based approaches to understanding phosphorus deprivation responses and PSR1 control in *Chlamydomonas reinhardtii. Eukaryot. Cell*. 5, 26–44 10.1128/EC.5.1.26-44.2006 16400166 PMC1360252

[B61] Moustafa A. Evans A. N. Kulis D. M. Hackett J. D. Erdner D. L. Anderson D. M. (2010). Transcriptome profiling of a toxic dinoflagellate reveals a gene-rich protist and a potential impact on gene expression due to bacterial presence. PLoS ONE 5:e9688 10.1371/journal.pone.0009688 20300646 PMC2837391

[B62] Ogawa N. DeRisi J. Brown P. O. (2000). New components of a system for phosphate accumulation and polyphosphate metabolism in *Saccharomyces cerevisiae* revealed by genomic expression analysis. Mol. Biol. Cell. 11, 4309–4321 10.1091/mbc.11.12.4309 11102525 PMC15074

[B63] Palenik B. Grimwood J. Aerts A. Rouzé P. Salamov A. Putnam N. (2007). The tiny eukaryote *Ostreococcus* provides genomic insights into the paradox of plankton speciation. Proc. Natl. Acad. Sci. USA. 104, 7705–7710 10.1073/pnas.0611046104 17460045 PMC1863510

[B64] Parsons T. R. Maita Y. Lalli C. M. (1984). A manual of Chemical and Biological Methods for Seawater Analysis. Oxford: Pergamon Press

[B65] Pinto E. Sigaud-Kutner T. C. S. Leitao M. A. S. Okamoto O. K. Morse D. Colepicolo P. (2003). Heavy metal-induced oxidative stress in algae. J. Phycol. 39, 1008–1018 10.1111/j.0022-3646.2003.02-193.x

[B66] Popels L. C. Hutchins D. A. (2002). Factors affecting dark survival of the brown tide alga *Aureococcus anophagefferens* (Pelagophyceae). J. Phycol. 38, 738–744 10.1046/j.1529-8817.2002.01115.x

[B67] Popels L. C. MacIntyre H. L. Warner M. E. Zhang Y. Hutchins D. A. (2007). Physiological responses during dark survival and recovery in *Aureococcus anophagefferens* (Pelagophyceae). J. Phycol. 43, 32–42 10.1111/j.1529-8817.2006.00303.x

[B68] Pride A. C. Herrera C. M. Guan Z. Giles D. K. Trent M. S. (2013). The outer surface lipoprotein VolA mediates utilization of exogenous lipids by *Vibrio cholerae*. MBio 4, e00305–e00313 10.1128/mBio.00305-13 23674613 PMC3656444

[B69] Rahman M. A. Hassler C. (2014). Is arsenic biotransformation a detoxification mechanism for microorganisms? Aquat. Toxicol. 146, 212–219 10.1016/j.aquatox.2013.11.009 24321575

[B70] Read B. A. Kegel J. Klute M. J. Kuo A. Lefebvre S. C. Maumus F. (2013). Pan genome of the phytoplankton *Emiliania huxleyi* underpins its global distribution. Nature 499, 209–213 10.1038/nature12221 23760476

[B71] Roberts A. Goff L. Pertea G. Kim D. Kelley D. R. Pimentel H. (2012). Differential gene and transcript expression analysis of RNA-seq experiments with TopHat and Cufflinks. Nat. Protoc. 7, 562–578 10.1038/nprot.2012.016 22383036 PMC3334321

[B72] Sanders J. G. (1985). Arsenic geochemistry in Chesapeake Bay: dependence upon anthropogenic inputs and phytoplankton species composition. Mar. Chem. 17, 329–340

[B73] Sanders J. G. Windom H. L. (1980). The uptake and reduction of arsenic species by marine algae. Estuar. Coast. Mar. Sci. 10, 555–567 24455932

[B74] Silver S. Phung L. T. (2005). Minireview: genes and enzymes involved in bacterial oxidation and reduction of inorganic arsenic. Appl. Env. Microbiol. 71, 599–608 10.1128/AEM.71.2.599-608.2005 15691908 PMC546828

[B75] Simpson J. T. Durbin R. (2012). Efficient *de novo* assembly of large genomes using compressed data structures. Genome Res. 22, 549–556 10.1101/gr.126953.111 22156294 PMC3290790

[B76] Simpson J. T. Wong K. Jackman S. D. Schein J. E. Jones S. J. M. Birol I. (2009). ABySS: a parallel assembler for short read sequence data. Genome Res. 19, 1117–1123 10.1101/gr.089532.108 19251739 PMC2694472

[B77] Stabenau H. Winkler U. Säftel W. (1989). Compartmentation of peroxisomal enzymes in algae of the group of prasinophyceae: occurrence of possible microbodies without catalase. Plant Physiol. 90, 754–759 16666839 10.1104/pp.90.2.754 PMC1061792

[B78] Sunda W. G. Granéli E. Gobler C. J. (2006). Positive feedback and the development and persistence of ecosystem disruptive algal blooms. J. Phycol. 42, 963–974 10.1111/j.1529-8817.2006.00261.x

[B79] Suzek B. E. Huang H. McGarvey P. Mazumder R. Wu C. H. (2007). UniRef: comprehensive and non-redundant UniProt reference clusters. Bioinformatics 23, 1282–1288 10.1093/bioinformatics/btm098 17379688

[B80] Thomas M. F. Abdul-Wajid S. Panduro M. Babiarz J. E. Rajaram M. Woodruff P. (2012). Eri1 regulates microRNA homeostasis and mouse lymphocyte development and antiviral function. Blood 120, 130–142 10.1182/blood-2011-11-394072 22613798 PMC3390952

[B81] Thompson A. W. Huang K. Saito M. A. Chisholm S. W. (2011). Transcriptome response of high- and low-light-adapted *Prochlorococcus* strains to changing iron availability. ISME J. 5, 1580–1594 10.1038/ismej.2011.49 21562599 PMC3176520

[B82] Toh-e A. Inouye S. Oshima Y. (1981). Structure and function of the PHO82-pho4 locus controlling the synthesis of repressible acid phosphatase of *Saccharomyces cerevisiae*. J. Bacteriol. 145, 221–232 7007314 10.1128/jb.145.1.221-232.1981 PMC217264

[B83] Van Mooy B. A. S. Fredricks H. F. Pedler B. E. Dyhrman S. T. Karl D. M. Koblížek M. (2009). Phytoplankton in the ocean use non-phosphorus lipids in response to phosphorus scarcity. Nature 457, 69–72 10.1038/nature07659 19182781

[B84] Veljanovski V. Vanderbeld B. Knowles V. L. Snedden W. A. Plaxton W. C. (2006). Biochemical and molecular characterization of AtPAP26, a vacuolar purple acid phosphatase up-regulated in phosphate-deprived *Arabidopsis* suspension cells and seedlings. Plant Physiol. 142, 1282–1293 10.1104/pp.106.087171 16963519 PMC1630754

[B85] Wilson P. A. (2006). Characterization of the human patatin-like phospholipase family. J. Lipid Res. 47, 1940–1949 10.1194/jlr.M600185-JLR200 16799181

[B86] Wu Z. Jenkins B. D. Rynearson T. A. Dyhrman S. T. Saito M. A. Mercier M. (2010). Empirical bayes analysis of sequencing-based transcriptional profiling without replicates. BMC Bioinformatics 11:564 10.1186/1471-2105-11-564 21080965 PMC3098101

[B87] Wurch L. L. Bertrand E. M. Saito M. A. Van Mooy B. A. S. Dyhrman S. T. (2011a). Proteome changes driven by phosphorus deficiency and recovery in the brown tide-forming alga *Aureococcus anophagefferens*. PLoS ONE 6:e28949 10.1371/journal.pone.0028949 22194955 PMC3237563

[B88] Wurch L. L. Haley S. T. Orchard E. D. Gobler C. J. Dyhrman S. T. (2011b). Nutrient-regulated transcriptional responses in the brown tide-forming alga *Aureococcus anophagefferens*. Environ. Microbiol. 13, 468–481 10.1111/j.1462-2920.2010.02351.x 20880332 PMC3282463

[B89] Wurch L. L. Gobler C. J. Dyhrman S. T. (2014). Expression of a xanthine and phosphate transporter in cultures and field populations of the harmful alga Aureococcus anophagefferens: tracking nutritional deficiency during brown tides. Environ. Microbiol. [Epub ahead of print]. 10.1111/1462-2920.12374 24373102

[B90] Wykoff D. D. Grossman A. R. Weeks D. P. Usuda H. Shimogawara K. (1999). Psr1, a nuclear localized protein that regulates phosphorus metabolism in Chlamydomonas. Proc. Natl. Acad. Sci. U.S.A. 96, 15336–15341 10611385 10.1073/pnas.96.26.15336 PMC24820

[B91] Zakharyan R. A. Tsaprailis G. Chowdhury U. K. Hernandez A. Aposhian H. V. (2005). Interactions of sodium selenite, glutathione, arsenic species, and omega class human glutathione transferase. Chem. Res. Toxicol. 18, 1287–1295 10.1021/tx0500530 16097802

[B92] Zhang Q. C. Qiu L. M. Yu R. C. Kong F. Z. Wang Y. F. Yan T. (2012). Emergence of brown tides caused by Aureococcus anophagefferens Hargraves et Sieburth in China. Harmful Algae 19, 117–124 10.1016/j.hal.2012.06.007

[B93] Zhang Z. Wood W. I. (2003). A profile hidden Markov model for signal peptides generated by HMMER. Bioinformatics 19, 307–308 10.1093/bioinformatics/19.2.307 12538263

